# Directed evolution for soluble and active periplasmic expression of bovine enterokinase in *Escherichia coli*

**DOI:** 10.1038/s41598-022-22574-6

**Published:** 2022-10-21

**Authors:** Weiluo Lee, Subhas Pradhan, Cheng Zhang, Niccolo A. E. Venanzi, Weina Li, Stephen Goldrick, Paul A. Dalby

**Affiliations:** 1 Department of Biochemical Engineering, UCL, London, WC1E 6BT UK; 2 EPSRC Future Targeted Healthcare Manufacturing Hub, UCL, London, WC1E 6BT UK; 3 Shaanxi R&D Center of Biomaterials and Fermentation Engineering, School of Chemical Engineering, Northwest University, 229 North Taibai Road, Xi’an, 710069 Shaanxi China

**Keywords:** Protein design, Biocatalysis, Proteases

## Abstract

Bovine enterokinase light chain (EK_L_) is an industrially useful protease for accurate removal of affinity-purification tags from high-value biopharmaceuticals. However, recombinant expression in *Escherichia coli* produces insoluble inclusion bodies, requiring solubilisation, refolding, and autocatalytic activation to recover functional enzyme. Error-prone PCR and DNA shuffling of the EK_L_ gene, T7 promoter, *lac* operon, ribosome binding site, and pelB leader sequence, yielded 321 unique variants after screening ~ 6500 colonies. The best variants had > 11,000-fold increased total activity in lysates, producing soluble enzyme that no longer needed refolding. Further characterisation identified the factors that improved total activity from an inactive and insoluble starting point. Stability was a major factor, whereby melting temperatures > 48.4 °C enabled good expression at 37 °C. Variants generally did not alter catalytic efficiency as measured by *k*_cat_/*K*_m_, which improved for only one variant. Codon optimisation improved the total activity in lysates produced at 37 °C. However, non-optimised codons and expression at 30 °C gave the highest activity through improved protein quality, with increased *k*_cat_ and *T*_m_ values. The 321 variants were statistically analysed and mapped to protein structure. Mutations detrimental to total activity and stability clustered around the active site. By contrast, variants with increased total activity tended to combine stabilising mutations that did not disrupt the active site.

## Introduction

Enterokinase, or enteropeptidase (EC 3.4.21.9), was the first known enzyme to activate other enzymes^1^. Porcine^2^, bovine^3^, and human^4^, enterokinases consist of an 82–140 kDa heavy chain and a 35–63 kDa light chain linked via a disulphide bond, with the range of molecular weights dependant on glycosylation patterns. The heavy chain is primarily involved in membrane association within the duodenum^5^ and trypsinogen recognition^6^ while the light chain is the catalytic serine protease^7^ that activates trypsinogen into trypsin through cleavage of an N-terminal hexapeptide Val-(Asp)_4_-Lys^8^. The light chain retains the specific activity characteristics of the heterodimer and is sufficient for specificity and activity towards the Val-(Asp)_4_-Lys peptide sequence^8^. This has made the enterokinase light chain (EK_L_) attractive for the controlled release of synthetic gene products, such as the specific cleavage of affinity-tags after protein purification^9,10^, particularly as the peptide cleavage does not leave any unwanted amino acid residues from affinity tags on the N-terminus of the target protein^8^.

EK_L_ was previously purified from porcine^2^ and bovine^3^ duodenums, and then recombinantly^11^ by fusion of the EK_L_ cDNA to the pre/pro region of human PACE, and expression in mammalian cells. EK_L_ is active when either glycosylated or non-glycosylated, and so the recombinant expression of both forms have been widely investigated, in heterologous hosts such as *E. coli*^12,13^, filamentous fungus *Aspergillus niger*^14^, methylotrophic yeast *Pichia pastoris*^15,16^, brewer’s yeast *Saccharomyces cerevisiae*^17^, dairy yeast *Kluyveromyces lactis*^18^, and insect baculovirus cells^19^.

Recombinant expression of EK_L_ in *E. coli* remains particularly problematic despite its speed, ease, and economy. The eukaryotic enzyme is natively glycosylated and requires disulphide-bond formations that are not efficiently catalysed in the *E. coli* cytoplasm. Thus it forms inclusion bodies that require in vitro solubilisation and refolding in order to recover the functional enzyme^20,21^. Improvements have often focused on using fusing the enzyme to solubility-enhancing proteins such as glutathione S-transferase (GST)^20^, thioredoxin^21,22^, protein disulphide isomerase (PDI)^23^, and DsbA^12,24,25^. In all of these cases, the fusion protein has required subsequent autocatalytic cleavage to regenerate the native N-terminus for EK_L_ and so its biological activity. In some instances, the EK_L_ fusion proteins still expressed predominantly as inclusion bodies, necessitating both refolding and autocatalytic cleavage steps to recover the functional enzyme^20,22^. Protein engineering efforts have also improved the yield of EK_L_ refolding from an insoluble fusion protein, such as by supercharging the surface of human EK and also removing a free cysteine (C112) by mutation to serine^22,26^.

Directed evolution has previously improved the soluble expression of eukaryotic proteins in *E. coli*^27,28^ as well as enhanced their secretion^29–31^. Here, we have exploited a combination of rational mutation, directed evolution and codon optimisation, to modify an expression system for Chinese yellow EK_L_ in *E. coli* that currently produces only insoluble inclusion bodies. Genetic mutations have the potential to influence many factors that could enable and improve the functional expression of EK_L_. These include protein properties, such as catalytic activity, solubility, aggregation propensity, conformational stability, folding and unfolding rates, as well those influencing transcription, translation and translocation rates. Therefore, we targeted random mutagenesis and DNA shuffling throughout the entire EK_L_ gene, and also in the T7 promoter, *lac* operator, ribosome binding site, and N-terminal pelB signal peptide, to generate variants of recombinant EK_L_ that are solubly and functionally expressed in the *E. coli* periplasm, as measured by their total activity in cell lysates. Analysis of 321 unique variants spanning a wide range of total activities, and further characterisation of selected purified variants identified the main factors that led to improved total activity when starting from an inclusion body forming protein.

## Materials and methods

Unless otherwise specified, all reagents and materials were purchased from Sigma-Aldrich UK Ltd (Dorset, UK).

### Cloning and site-directed mutagenesis

The parent template gene of the Chinese yellow bovine EK_L,_ Cny-EK_L_, sequence (UniProtKB Q6B4R4), was cloned into pET26b + (Novagen, UK), which also added an N-terminally fused pelB secretion peptide, and C-terminally fused His_6_ tag (Supplementary Fig. S1). Site-directed mutagenesis used the QuikChange II site-directed mutagenesis kit (Agilent Technologies, UK) as per the manufacturer’s instructions, and mutagenic primers from Eurofins Genomics (Ebersberg, Germany) (Table 1), with transformation by electroporation into either *E. cloni* EXPRESS BL21(DE3) or OverExpress C41(DE3) cells (Lucigen, UK). Transformants were plated on LB agar (+ 40 μg mL^−1^ kanamycin) and incubated at 37 °C overnight. Colonies were picked and grown in 5 mL LB with 40 μg mL^−1^ kanamycin, and used to generate glycerol stocks for storage at − 70 °C.

**Table 1:** Mutant primers for site-directed mutagenesis.

Mutation	Primer Sequence (5'–3')	Mutation type
R82P	ccaaatagtaattaat**ccg**cactataacaaac	Present in bovine EK (UniProtKB P98072) & Consensus
V15Q	cgtgagggggcatggccgtgg**cag**gtggctctctattttgatgatc	Consensus
A17S	gagggggcatggccgtgggtggtg**tct**ctctattttgatgatcaacagg	Consensus
A28G	atcaacaggtctgcggc**ggc**tctttagtgtctcgtgattggctgg	Consensus
V43F	ctggtgagcgcagcgcactgt**ttt**tacggccgtaacatggaaccgtcaaaatgg	Consensus
A55V	ggaaccgtcaaaatggaag**gta**gtactggggttgcacatggccagc	Consensus
C112S	ggattacattcaaccaatt**tct**ctgccggaagaaaacc	Improved in vitro folding^45^

### Activity detection of variants

Preliminary activities for the consensus variants of Cny-EK_L_, were determined as described in the supplementary information (Sect. S3). All further assays were performed as below for increased sensitivity. The total activity of EK_L_ variants was determined after expression by fermentation in 96-microwell plates. Individual colonies were picked manually into shallow, round-bottom 96-microwell plates (Starstedt, UK) containing 150 μL per well LB with 40 μg mL^−1^ kanamycin, then sealed with breathable film (Axygen, USA), and incubated at 30 °C, 85% humidity for 16–20 h with 400 rpm shaking at 25 mm throw in an ISF-1-W microwell shaker (Kuhner, UK). Samples of 10 μL per well were then used to inoculate deep, V-bottom 96-microwell plates (Axygen, USA) containing 390 μL TB per well with 40 μg mL^−1^ kanamycin, sealed with breathable film and incubated as above for 6 h, to an OD_600_ of 0.5–1, then induced at 0.2 mM IPTG by dispensing 10 μL 8 mM IPTG per well, and incubated for a further 4 h. Alternatively, for expression at 37 °C, pre-induction and post-induction growth times were 4.5 h and 4 h, respectively. Expression plates were harvested by centrifuging at 4000 rpm, 4 °C in an Eppendorf centrifuge 5810R (Eppendorf AG, UK) for 10 min and the spent media decanted.

A 400 μL aliquot of lysis mixture (1 mg mL^−1^ lysozyme, 0.5 mg mL^−1^ polymyxin B sulphate (PMBS; AppliChem GmbH, UK) in 70 mM Tris–HCL, 50 mM NaCl, pH 8.4, 1 mM EDTA), was added to each well containing the cell pellets. Plates were sealed with clear film (Axygen, UK) and shaken vigorously in plate shakers (Thermo Fisher Scientific, UK) for 2 h. Cellular debris was pelleted by centrifuging at 4000 rpm, 4 °C in an Eppendorf centrifuge 5810R for 10 min, and 20 μL of each clarified lysate transferred into wells of a shallow black fluorescence 96-microwell assay plates (Thermo Scientific Nunc, UK) containing 170 μL reaction buffer per well. Cleavage of the GD4K-na fluorogenic substrate was monitored using a FLUOstar OPTIMA microwell plate spectrophotometer (BMG Labtech, UK) with 340 nm excitation and 420 nm emission to obtain initial rates. Reactions were initiated by auto-injection of 10 μL of 1.25 mM GD4K-na substrate per well, pre-dissolved in the reaction buffer with 1 mM EDTA, giving final reaction conditions of 0.0625 mM GD4K-na, 70 mM Tris–HCl, 50 mM NaCl, 1 mM EDTA, pH 8.4 at 25 °C.

### Random mutagenesis and DNA-shuffling libraries

Random mutagenesis was carried out using the GeneMorph II EZClone Domain Mutagenesis kit (Agilent Technologies, UK) with the manufacturer’s instructions. The forward (5’-cgtagaggatcgagatctcg-3’) and reverse (5’-gctagttattgctcagcgg-3’) primers (Eurofins Genomics, UK) for epPCR, flanked the region of the gene containing the T7 promoter, *lac* operator, ribosomal binding site (rbs), pelB signal peptide, EK_L_ gene, and His_6_ tag. Mutation rates were tuned by varying the parent plasmid concentration. The MEGAWHOP (megaprimer PCR of whole plasmid) step^32^ was carried out with the empty pET26b + vector instead of the parent plasmid containing the EK_L_ gene, and variants electroporated into *E.cloni* EXPRESS BL21(DE3).

Variants selected after screening of the random mutagenesis libraries were randomly recombined using DNA-shuffling^33^ with slight modifications. Briefly, DNA-shuffling was carried out with the following steps: (1) amplification of the parent template(s) using forward (5’-ggtgatgtcggcgatatagg-3’) and reverse (5’-ccgtttagaggccccaagg-3’) primers, (2) DNA fragmentation of the parent template(s), (3) DNA assembly either with or without additional mutagenic primers, (4) amplification of the assembled gene templates using forward (5’-cgtagaggatcgagatctcg-3’) and reverse (5’-ccgtttagaggccccaagg-3’) primers, (5) MEGAWHOP cloning into the empty pET26b + vector, and (6) DpnI (New England Biolabs, UK) digestion of the pET26b + vector and then transformation into *E. coli*. In instances where interesting variant templates were DNA-shuffled, multiple parent templates were amplified in step 1 and DNA assembly in step 3 was performed with or without additional mutagenic primers as required. dUTP was added alongside dNTPs, for random incorporation of uracil into gene sequences. Pfu Turbo Cx Hotstart DNA polymerase (Agilent Technologies, UK) replaced Taq polymerase, to facilitate uracil incorporation while maintaining high-fidelity in steps 1, 3 and 4. Endonuclease V (New England Biolabs, UK) replaced DNAse I in step 2 to cleave the second and third phosphodiester bonds 3’ to the incorporated dUTP and more effectively fragment the parent template. High-fidelity Pfu Turbo DNA polymerase (Stratagene, UK) was used in the MEGAWHOP cloning step. Variants were transformed into OverExpress C41(DE3) electrocompetent cells, and plated on LB agar with 40 µg mL^−1^ kanamycin, in square bio-assay dishes (Thermo Scientific Nunc, UK) and incbated at 37 °C for 14–20 h to facilitate high-throughput colony-picking.

### High throughput screening

Activity detection of variants produced from random mutagenesis and DNA/HITS-shuffling libraries was carried out as described above, except that colony picking was automated with a QPix2 colony picker (Molecular Devices, UK), and liquid-handling was automated using a Freedom Evo TECAN (Reading, UK). BL21(DE3) or C41(DE3) harbouring the empty pET26b + vector were used in triplicate as negative controls in all library screens. The relevant parent variant in BL21(DE3) or C41(DE3) was used in triplicate as positive controls.

The cleavage of the GD4K-na fluorescence substrate was monitored as above, for the reaction plates sealed with aluminium thermowell sealing tape (Costar, UK) and incubated at 25 °C with no shaking. To additionally assess EK_L_ variant stabilities, the sealed microplates were pre-incubated in a 50 °C oven for 2 h, immediately cooled to 0 °C in a shallow ice bath, then warmed to 25 °C in the platereader prior to initiating the reaction by auto-injection of the substrate as described above. This condition was tuned to obtain a loss of activity of 50% in the parent EK, thus enabling both improved and destabilised variants to be quantified.

### Retest assay

Fermentation conditions and procedures were the same as stated for screening above, except that for 30 °C expression, pre-induction and post-induction growth times were 6 h and 4 h, respectively, while for 37 °C expression, pre-induction and post-induction growth times were 4.5 h and 4 h, respectively. Lysis and assay conditions were the same as used in the screening above. In order to relate the spectrophotometer’s fluorescence unit (FU) signal to the amount of 2-naphthylamine fluorometric product liberated (or GD_4_K-na substrate cleaved) in mM units, a linear standard curve was produced to determine a “FU mM^-1^” conversion factor. As the 2-naphthylamine product is unavailable in the UK, the standard curve was obtained from complete conversion of a range of GD_4_K-na substrate concentrations using commercial EK_L_ (New England Biolabs, UK) in 70 mM Tris–HCl, 50 mM NaCl, pH 8.4, at 25 °C. Reactions were monitored until the FU signals plateaued, signifying complete conversion and the final FU values taken for the standard curve. Linear regression determined the FU mM^−1^ 2-naphthylamine conversion factor from the slope, as 7599 FU mM^−1^.

### Statistical analysis

A partial least squares (PLS) regression analysis was conducted in OriginPro 2018 (Origin Lab Corp., Northampton, MA, USA) using default settings, to estimate the effects of individual mutations (independent variables) within the 321 unique EK_L_ variants, on the four dependent (response) variables of total activity and residual activity after heating, when expressed at both 30 °C and 37 °C. Variants included nucleobase mutations found on the T7 promoter, *lac* operator, ribosomal binding site, pelB leader, and EK_L_ genes, as well as non-synonymous residue mutations within the pelB secretion peptide and EK_L_ protein. Redundant mutations were removed where multiple mutations always coexisted, and labelled as a single factor with multiple mutations (eg. I135S/t556g). The PLS analysis was applied simultaneously to all four responses to obtain a single model with a single variable importance in projection (VIP) value for each mutation, but separate coefficients used to predict each response for each mutation. The coefficients for each mutation were obtained from the optimal model in which tenfold cross-validation had determined the number of components that gave the minimum predicted residual sum of squares. The importance of each mutation in the model was determined from their VIP values.

### Protein expression and purification

EK_L_ variants were grown in 10 mL LB with 40 μg mL^−1^ kanamycin, for 16 h at 30 °C with 250 rpm shaking in an ISF-1-W shaker, and then 4 mL used to inoculate each of two 2-L non-baffled shake flasks containing 400 mL TB with 40 μg mL^−1^ kanamycin. Pre-induction and post-induction growth times were 6 h and 4 h, respectively for 30 °C expression, and 4.5 h and 4 h, respectively for 37 °C expression. Expression was initiated by adding 400 μL 200 mM IPTG to each shake flask, and the final cultures from the two 2-L shake flasks were combined. A 500 μL aliquot was taken for measurement of the total activity, and the remaining culture harvested by centrifugation at 10,000 rpm for 20 min at 4 °C in a J2-MI centrifuge (Beckman Coulter, UK). Cell pellets were re-suspended in 40 mL Ni-affinity binding buffer (20 mM sodium phosphate, 500 mM NaCl, 40 mM imidazole, pH 7.4), and chilled on ice for 15 min before sonication (10 s on, 10 s off intervals with 10 μm pulses; 20 cycles) using a Soniprep 150 sonicator (MSE, UK). Crude lysates were clarified at 10,000 rpm in a J2-MI centrifuge for 45 min at 4 °C, then 30 mL loaded onto HisTrapFF 5 mL columns (GE Healthcare, UK) pre-equilibrated with Ni-affinity binding buffer, using an AKTA purifier FPLC system. The column was washed with 10 column volumes of binding buffer, then eluted with 5 column volumes of 20 mM sodium phosphate, 500 mM NaCl, 500 mM imidazole, pH 7.4, and fractions (E1–E8) analysed as above for EK_L_ activity. For subsequent STI-affinity purification, a Tricorn 5/50 column (GE Healthcare, UK) was packed with ~ 1 mL of soybean trypsin inhibitor (STI) agarose and equilibrated with STI-affinity binding buffer (10 mM Tris–HCl, 500 mM NaCl, pH 8.0). Approximately 7–10 mL of the pooled active EK_L_ fractions from Ni-affinity purification were loaded onto the column and washed with 10 column volumes of the STI-affinity binding buffer, then eluted with 15 mL of 75 mM glycine, 500 mM NaCl, pH 3.0, and fractionated into deep, V-bottom 96-microwell plates containing 50 µL 2 M Tris–HCl pH 8.0 per well. Fractions containing active EK_L_ were pooled, then concentrated and buffer-exchanged into the reaction buffer (without EDTA) using a 10,000 MWCO VivaSpin 2 (Sartorius, UK). Protein concentrations were determined by absorbance at 280 nm on a NanoDrop 2000c (Thermo Scientific, UK), an extinction coefficient of 53,900 M^−1^ cm^−1^ based on amino acid sequence^34^, and MW of 26,260.7 Da. Purity was analysed by SDS-PAGE.

### Enzyme kinetics

Initial rates for purified EK_L_ variants were assessed in triplicate, at substrate concentrations, [S] for GD4K-na, of 0.00625, 0.0125, 0.01875, 0.025, 0.03125, 0.0625, 0.1125, 0.25, 0.4375, 0.625, 0.8125, and 1 mM. Stocks of substrate at 1.25 mM or 0.125 mM, and protein variants, in reaction buffers (without EDTA) were pre-warmed to 25 °C, then 20 μL per well of the EK_L_ variant stock solution added to the fluorescence assay plates, and then mixed well upon addition of appropriate volumes of reaction buffer. Reactions were initiated in the FLUOStar Optima by auto-injection of corresponding volumes of either 1.25 mM or 0.125 mM substrate stock solutions, and monitored at 25 °C. Initial rates (*v*) in triplicate were averaged before fitting to the Michaelis–Menten equation (Eq. 1) in OriginPro 2017, to obtain catalytic turnover rates (*k*_cat_), Michaelis–Menten constants (*K*_m_), and substrate inhibition constants (*K*_s_). The standard curve of slope 7599 FU/mM described above was used to make the appropriate unit conversions.

1 $$ v = { }\frac{{V_{max} }}{{\frac{{K_{m} }}{{\left[ {\text{S}} \right]}}{ } + 1 + { }\frac{{\left[ {\text{S}} \right]^{n - 1} }}{{K_{i}^{n - 1} }}}} $$

Total activity from the 500 μL shake flask aliquots, was also determined as above, and then the soluble expression levels of functional enzymes were estimated by dividing the total activity of the variants measured in lysates at 0.0625 mM substrate, by their specific activity (initial velocity/enzyme concentration) as determined using the same purified variant with 0.0625 mM substrate. Determination of the soluble expression level was also estimated directly from SDS-PAGE for some variants.

### Thermostability

The thermal transition midpoint temperatures (*T*_m_) of 0.1 mg mL^−1^ purified EK_L_ variants in 70 mM Tris–HCl, 50 mM NaCl, pH 8.4, were measured in triplicate, with an Optim 1000 (Unchained Laboratories, UK). Intrinsic fluorescence from 280 nm excitation was measured during stepped thermal ramping from 25 to 95 °C at a 1 °C min^−1^ ramp rate. *T*_m_ values were obtained from the protein melting curves using the Optim Analysis software v2.0 (Unchained Laboratories, UK).

Unfolding rates at 45 °C (*k*_u,45 °C_) were also measured in triplicate under isothermal conditions, from the rate of change in intrinsic fluorescence over 24 h, using the Optim 1000, and 0.1 mg mL^−1^ purified EK_L_ variants in 70 mM Tris–HCl, 50 mM NaCl, pH 8.4. The raw data was imported into OriginPro 2018 and curve-fitted to Eq. 2:

2 $$ {\text{y }} = {\text{ y}}_{0} + {\text{ Ae}}^{{k}{.{\text{t}}}} $$

where *k* is the unfolding rate constant at 45 °C, *k*_u,45 °C_.

## Results and discussion

### Strain construction, rational mutagenesis, and culture temperature selection

The pelB secretion peptide^35^ was fused to the N-terminus of the mature Chinese northern yellow Cny-EK_L_ (UniProtKB Q6B4R4) EK_L_ protein sequence (Supplementary Fig. S1), in an attempt to secrete EK_L_ into the periplasm and thus facilitate cleavage of the pelB signal peptide, folding of the mature EK_L_ protein, and disulphide-bond formation. Even after optimising the fermentation parameters with *E. coli* BL21(DE3), the best conditions at 30 °C still gave poor soluble expression (0.5 mg L^−1^ or 0.13 mg L^−1^ OD_600_^–1^), detectable by SDS-PAGE only after concentrating the cells 20-fold by centrifugation and resuspension, while 99% of the protein formed inclusion bodies (> 55 mg L^−1^ or 13 mg L^−1^ OD_600_^–1^). A very low level of activity was detected using a fluorescent assay, also after the 20-fold concentration of cells, and osmotic shock to release the periplasmic fraction. A multiple sequence alignment of 250 sequences retrieved in a BLAST search, identified point mutations towards the consensus sequence with the potential to improve folding, stability, and (we hypothesised) soluble expression (Supplementary Fig. S2, and Table S1). Five consensus mutations were constructed, and while four improved soluble expression to some degree (Supplementary Fig. S3a), V15Q was found to give the greatest improvement with double the soluble expression, while retaining significant activity (Supplementary Fig. S3b). The soluble periplasmically secreted protein remained in the range 0.8–2.5% of total protein for all variants, with the remainder expressed as inclusion bodies.

The more widely studied Bovine EK_L_ (UniProtKB P98072) differs from Cny-EK_L_ via only two mutations R82P and D176E. R82P was also a consensus mutation (Supplementary Table S1). Previous work has shown that these mutations do not interfere with inclusion body formation or subsequent solubilisation, in vitro refolding, and autocatalytic activation^20^. The C112S mutation was also previously found to enhance in vitro refolding yields from solubilised inclusion bodies by 50%^22^. We hypothesised that the C112S and consensus R82P mutations could potentially improve EK_L_ folding in vivo. Therefore, we introduced the V15Q, R82P, and C112S mutations, to compare the eight variants (WT pelB-CnyEK_L_, V15Q, R82P, C112S, V15Q/R82P, R82P/C112S, V15Q/C112S, and V15Q/R82P/C112S). These were expressed in TB medium at both 30 °C and 37 °C in *E. coli* BL21(DE3), and also in C41(DE3) as this was reported to be more tolerant to toxic protein expression^36^. The variants were then compared for their total activity within lysates, using a more sensitive version of the fluorescence assay that avoided the need to pre-concentrate the cells.

**Figure 1:**
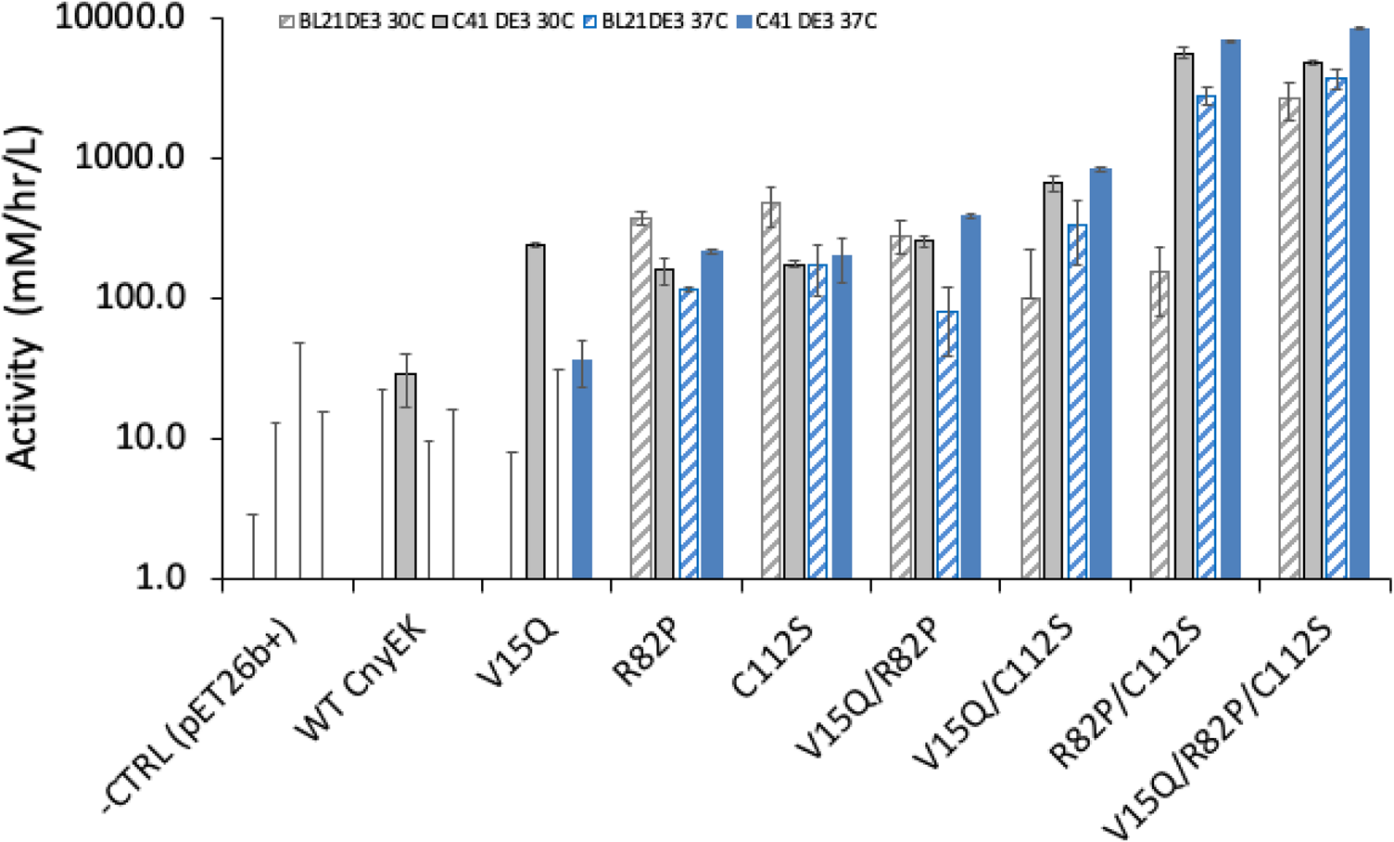
Total activity of EK_L_ variants at 30° or 37 °C in *E. coli* strains BL21(DE3) or C41(DE3). Cells expressing the pelB-fused Chinese northern yellow Cny-EK_L_ variants were grown at 37 °C or 30 °C for 16–20 h in shallow round-bottom 96-microwell plates, then again after dilution in deep V-bottom 96-microwell plates, for 6 h, induced with IPTG, and incubated for a further 4 h. Cells were pelleted and resuspended in a lysis mixture, then clarified, before assaying for EK_L_ total activity with the GD4K-na fluorogenic substrate at pH 8.4, 25 °C.

The relative total activity at 30 °C and 37 °C for each variant-strain combination, is shown in Fig. 1. *E. coli* C41(DE3) and 37 °C gave consistently higher total activity than BL21(DE3) and 30 °C respectively, for the best variants. The activity of WT pelB-CnyEK_L_ expressed at 37 °C could not be detected above the control background in either strain, whereas a low activity was detectable when expressed at 30 °C, though only in C41(DE3). V15Q gave an 8.5-fold increase in this activity to 240 mM hr^−1^ L^ 1^, and also detectable activity when expressed at 37 °C in C41(DE3). By contrast, R82P and C112S each gave a similar improvements in activity across all conditions, from 160–210 mM hr^−1^ L^−1^, though with slightly higher activities of 370 and 470 mM hr^−1^ L^−1^ respectively, for BL21(DE3) expression at 30 °C. The addition of V15Q within V15Q/R82P gave only modest changes in total activity, with slight increases in C41(DE3) and slight decreases in BL21(DE3). By contrast the addition of C112S within R82P/C112S led to a 31-fold improvement in activity in C41(DE3) at 37 °C, compared to R82P, and a 24-fold improvement in BL21(DE3) at 37 °C. Comparisons of single mutations when forming double, and triple mutants revealed the same general impacts of the individual mutations as modest (1.2–3.8 fold increases) for V15Q, significant for R82P (5–78 fold increases), and significant for C112S (16 to 40-fold increases). The highest activity attained was for V15Q/R82P/C112S at 37 °C in C41(DE3), with a 230-fold improvement over that of V15Q, and at least 340-fold higher than the WT CnyEK_L_, assuming it had activity just below the minimum detection limit of 15 mM hr^−1^ L^−1^.

That the R82P mutation was highly beneficial was consistent with both the expectation that consensus mutations are frequently stabilising^37^, and also that proline residues are known to be often stabilising, particularly to loop structures^38^. The R82P mutation is located within a large structured loop (residues Asn81-Ile94) that also forms part of the active site.

Residue C112 of EK_L_ is is unpaired in recombinant expressions of only the light chain, and a previous study had shown that the C112S mutation was shown to improve refolding yields by 50% for human EK_L_^22^, so it is likely also to have contributed positively through a similar mechanism in vivo here.

### Random mutagenesis

It was fully expected that DNA-level mutagenesis of the T7 promoter, *lac* operator, and rbs, as well as silent mutations within the pelB and EK_L_ sequences, could alter the expression dynamics and hence functional expression levels achieved. Therefore, random mutagenesis and DNA-shuffling techniques were targeted across a range of gene elements consisting of the T7 promoter, *lac* operator, ribosome binding site (rbs), pelB signal peptide, and the EK_L_ gene sequence (Supplementary Fig. S1).

**Figure 2:**
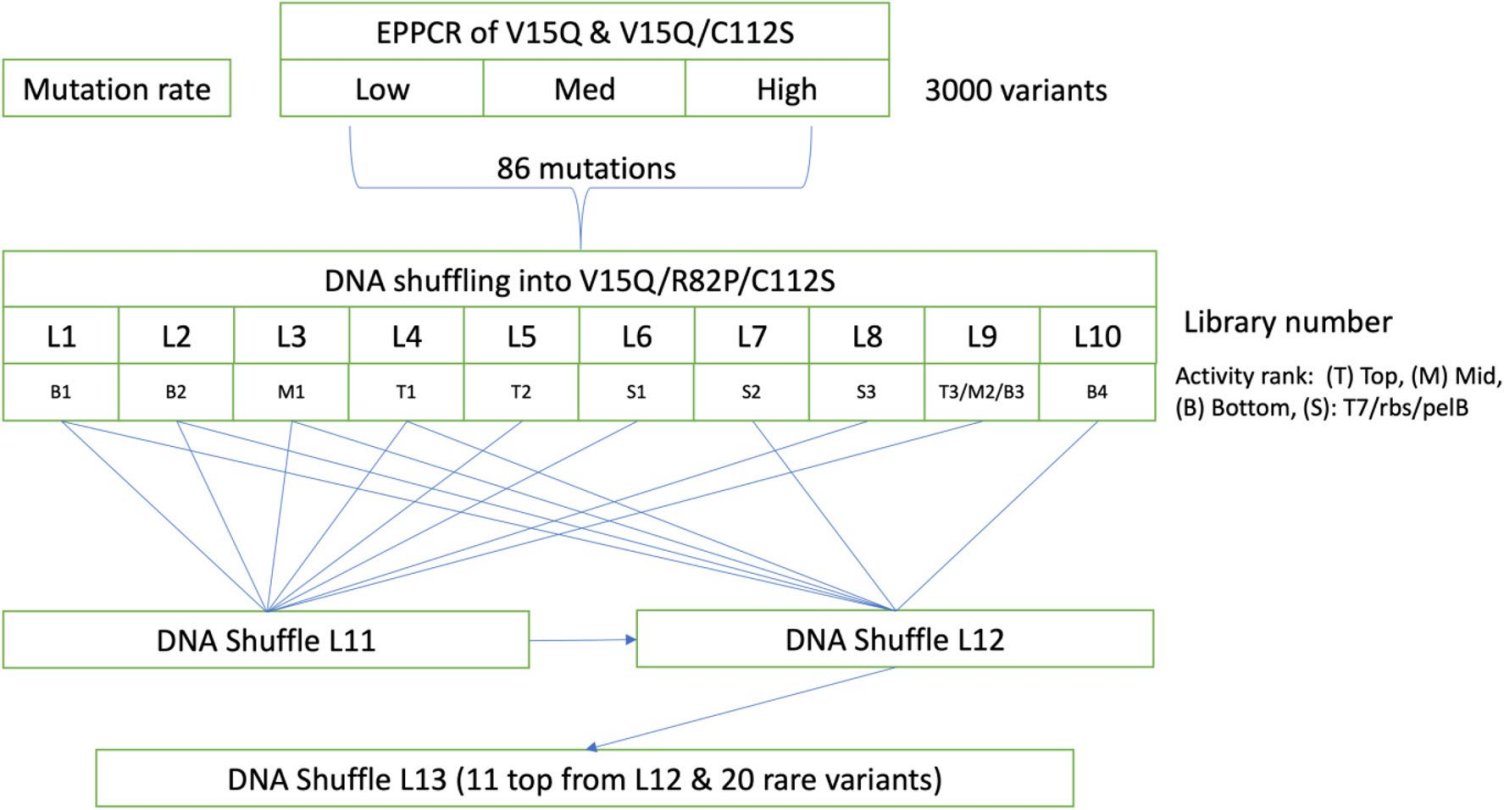
Schematic of overall library generation and iteration strategy. Error-prone PCR (EPPCR) generated the initial 3000 variants from libraries with three different mutation rates. DNA shuffling of the 86 identified and ranked mutations led to libraries L1 to L10. Further DNA shuffling of top hits from libraries L1 to L10 led to libraries L11 and L12. Library L13 was then generated from the 11 top hits from L12, plus 20 rare variants from across all libraries. Each library (L1, L2 etc.) is described in more detail in Tables S2, S3 and S4.

The overall library generation strategy, starting with random mutagenesis, is shown in Fig. 2. Random mutagenesis by error-prone PCR was applied first on the parent variants V15Q and V15Q/C112S, providing an evolutionary option to discover beneficial mutations that could match, or simply rediscover, the beneficial effect of the R82P mutation. Variants were also screened in *E. coli* BL21(DE3) at 30 °C, to discover mutations that could be potentially beneficial under conditions where the total activity was lowest and yet still measurable using high throughput plate-based growth and assays. Thereafter, iterations of DNA-shuffling and HITS-shuffling were performed to optimize the mutational compositions of evolved EK_L_ variants. For shuffling, more moderate evolutionary pressures were applied by expressing the enzyme variants from *E. coli* C41(DE3) at 30 °C. The stabilities of variants to thermal inactivation were also assessed via the loss of activity after a heat challenge, though this was not used as a screening filter. Therefore, EK_L_ variants exhibiting a wide range of improved total activities and stabilities were discovered throughout the directed evolution campaign.

Three random mutagenesis libraries were constructed and screened. A mutation rate of typically 1–3 base changes, with some containing as many as six base changes, was obtained. Given the high level of *E. coli* background protease activity towards the GD4K-na substrate, compared to the low total activity of EK_L_ variants, all variants with at least 1.2-fold improved activities over their respective parents (V15Q and V15Q/C112S) were included in subsequent DNA-shuffling rounds. Of approximately 3000 random mutagenesis variants screened, 225 EK_L_ variants were found to have improved total activity, and were therefore sequenced and mined for beneficial mutations. A total of 86 mutations were discovered and taken forward to construct DNA-shuffling libraries.

### First-round DNA-shuffling libraries

DNA-shuffling of all 86 variants would constitute a library size of 2^86^ possible variants. Instead, several smaller libraries that shuffled typically only 2–6 variants in each, aimed to maintain manageable library sizes and ensure that each mutation was represented at least once in the screening. Ten DNA-shuffled combinatorial libraries (L1–L10) were constructed on the V15Q/R82P/C112S parent variant, which re-introduced the beneficial R82P mutation, and then transformed into the more favourable C41(DE3) *E. coli* strain. The mutagenic primers incorporated into libraries (L1–L10) are shown in Supplementary Table S2. The possible number of variants in each library (except L10) varied from 4 to 64, and so each was screened within one or two 96-microwell plates while retaining a 95% probability of observing each variant at least once^39^. Variants containing non-synonymous mutations were first grouped as bottom, middle, and top-tiered depending on their relative total activity, and subsets of each tier shuffled into libraries L1–L5 as in Table S2. Additionally, variants with mutations residing in expression or secretion-related gene elements such as the T7 promoter, *lac* operator, and the ribosome binding site were independently shuffled in libraries L6–L8. Finally, libraries L9 and L10 consisted of the remaining mutations that were derived from randomly mutagenized variants that were at least 1.2 × improved over V15Q or V15Q/C112S. Sequencing confirmed the presence of 1–6 mutations in each new variant.

**Figure 3:**
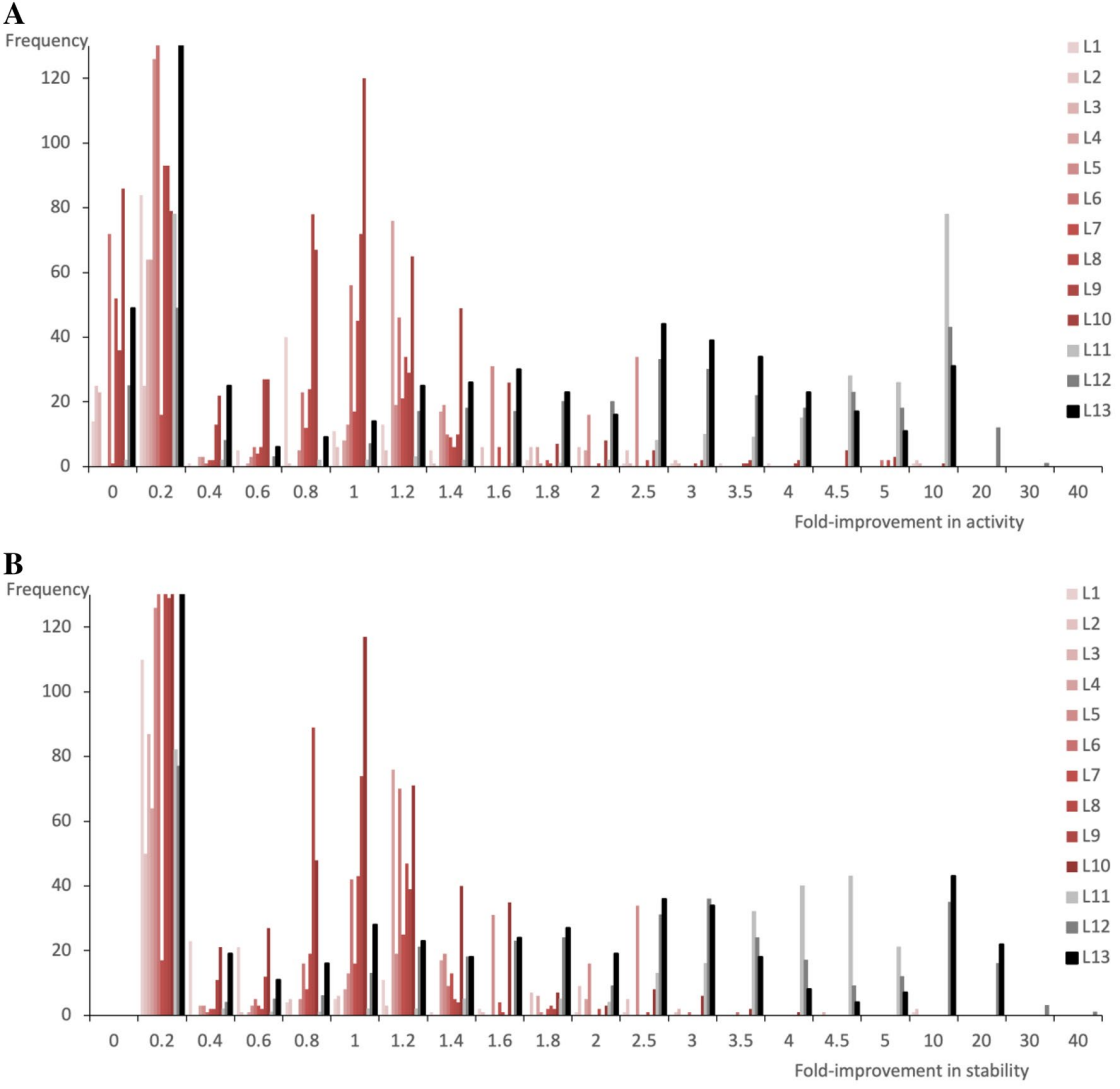
Distribution of improvements in (**A**) activity and (**B**) stability from each shuffling library (L1–L13). First-round shuffling libraries L1–L10 are shown in red shades, and rarely led to variants with > twofold improvement in activity or stability. Second to fourth-round shuffling libraries L11–L13 are shown in black shades. Each progressively led to more variants with higher (> 2.5-fold) activity and stability improvements. All improvements are relative to the V15Q/R82P/C112S parent. Y-axes are clipped at frequency = 130 in both plots to clearly visualise populations with > 0.4-fold improvement.

Approximately 2400 variants were screened in total from across libraries L1–10, and 370 variants with up to tenfold improvements in total activity, and up to fivefold improvement in residual activity after heat inactivation compared to the V15Q/R82P/C112S parent (Fig. 3), were selected and sequenced. The earlier strategy of randomly-mutagenizing debilitated EK_L_ parent variants containing the P82R mutation, and their expression in the BL21(DE3) strain that was less tolerant to functional EK_L_ expression and secretion, thus provided appropriate evolutionary pressures to discover beneficial mutations, that subsequently also recombined beneficially.

### Second to fourth-round DNA-shuffling

Top variants selected from the first-round DNA-shuffling libraries were further shuffled in three new successive libraries (Supplementary Tables S3 and S4). New variants were selected based on a combination of improved total activity and also stability as measured by residual activity after heating. As unintended random mutations were also introduced during the construction of the first round of DNA-shuffling libraries, with unclear contributions, mutant primers based on these mutations were also designed and included in one of the three variant-shuffling libraries. Library L11 was generated by shuffling the two highest activity variants from each of the eight libraries L1–6, L8 and L9 (Supplementary Table S3). Library L12 was generated by shuffling ten additional high-activity variants from libraries L1–L4, L7, and L10, plus one newly-discovered variant from library L11 (Table S3). Library L13 was then generated by shuffling eleven top variants for activity from library L12, along with short oligos encoding twenty less frequently observed mutations from libraries (L1–L12) (Supplementary Table S4), plus six newly appearing random mutations arising from DNA shuffling libraries (L1–L10), with the aim of maximising the diversity search. Approximately 1200 variants were screened (270 from L11, 360 from L12, 540 from L13) and 251 variants sequenced (61 from L11, 86 from L12, 104 from L13). Two separate EK_L_ variants were found to be improved up to 24-fold in total activity and up to 37-fold in stability over the V15Q/R82P/C112S parent variant, respectively (Fig. 3). While both of these top two variants were obtained from library L12, the final library L13 contained a higher frequency of variants with increased stability. The results also showed that the combinatorial libraries (L11–L13) were successful in further increasing the genetic diversity between the top hits having improved total activity.

### Retesting of all improved variants

**Figure 4:**
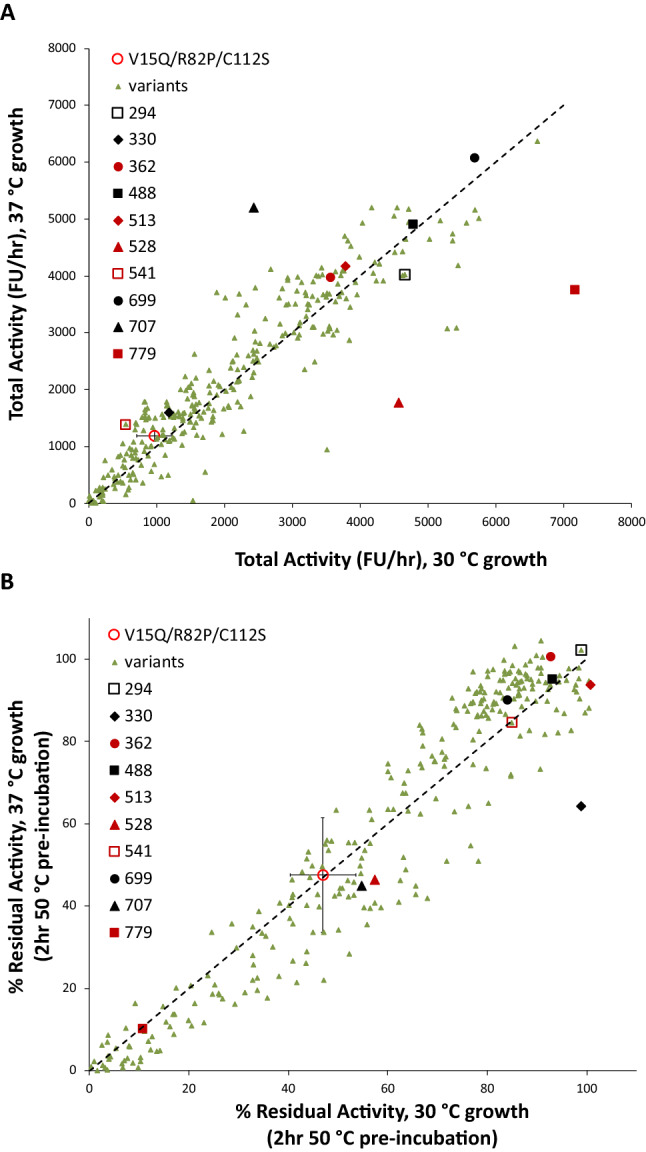
Total activities and stabilities of 321 EK_L_ variants from retest assays. V15Q/R82P/C112S (red open circle with standard deviation error bars) was assayed in triplicate whereas the rest were assayed in singlicates. (**A**) Comparison of total activities for the variants expressed at 30 °C and 37 °C. (**B**) Comparison of residual activities after a 2-h heat inactivation at 50 °C, for the variants expressed at 30 °C and 37 °C. Interesting EK_L_ variants are highlighted with larger symbols and noted in the figure legend.

Overall, approximately 6500 EK_L_ variants, derived from three random mutagenesis and thirteen combinatorial shuffling libraries, were evolved primarily for improved total activity while also measuring the stability of evolved variants to thermal inactivation. From the thirteen combinatorial library screens, 846 EK_L_ variants with a range of total activity and stability phenotypes were sequenced. It was found that 321 of the 846 sequenced EK_L_ variants were unique (Supplementary Table S5). These unique EK_L_ variants were retested together to enable a direct comparison of their total activity and stability. Retests were carried out with EK_L_ variants expressed at both 30 °C and 37 °C to investigate their sensitivity to expression temperature. Comparisons of the total activity and stability to thermo-inactivation when expressed at each temperature, are shown in Fig. 4, for all unique variants. Interesting variants representing a range of properties were selected for further characterisation below, and are highlighted with larger symbols, including V15Q/R82P/C112S as a reference point. The evolution of total activity for key variants throughout this work is summarised also in Supplementary Fig. S4.

Particularly interesting variants included WLEK0362, and WLEK0699, and WLEK0779. WLEK0362 was one of the most stable variants with 93–100% retained activity after heat inactivation, compared to just 4.3% for V15Q/R82P/C112S and 0% for V15Q. It also had moderately improved total activity compared to V15Q/R82P/C112S (1.8-fold at 30 °C and 1.4-fold at 37 °C), but was significantly improved over the original WT Cny-EK_L_ (310-fold at 30 °C and 11,300-fold at 37 °C). Indeed it had the highest overall total activity when expressed at 37 °C. This rise to become the best variant at 37 °C was likely due to the WLEK0362 variant being also the most stable, aiding its high soluble and functional expression. WLEK0699 exhibited both good stability (84–89% retained activity) and high total activity that was up to 3.7-fold improved over V15Q/R82P/C112S, and 620-fold higher than WT Cny-EK_L_ when expressed at 30 °C. By contrast, WLEK0779 exhibited the highest total activity when expressed at 30 °C (fourfold higher than V15Q/R82P/C112S, and 680-fold higher than WT Cny-EK_L_), but was only 50% as active when expressed at 37 °C. Heat inactivation resulted in only 10% residual activity for WLEK0779, and so its poor stability was likely to have been the reason for activity loss when expressed at 37 °C.

The expression temperature did not significantly effect the total activity for most EK_L_ variants (Fig. 4A), although a small number of outliers were highly sensitive to expression temperature. However, the expression temperature did result in a noticeable drop in stability to thermo-inactivation for many variants expressed at 37 °C compared to at 30 °C. This mainly affected the less stable variants, indicating that they had already become more susceptible to thermal denaturation during expression at the higher temperature, perhaps through formation of a population of partially denatured protein or soluble aggregates, that promote further unfolding or aggregation. A parity in stability was reached for the more stable variants, indicating sufficient stability to tolerate expression at 37 °C.

**Figure 5:**
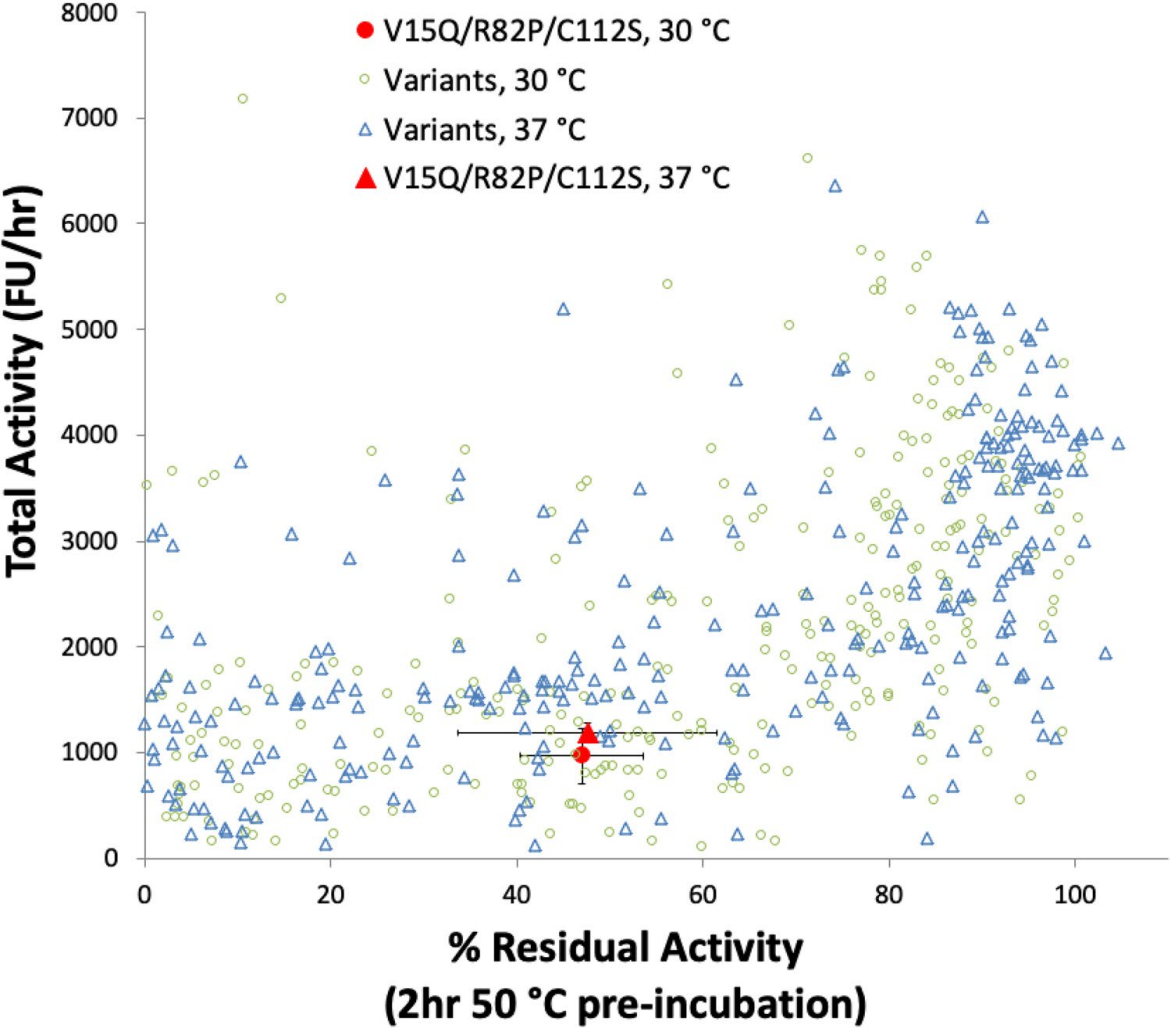
Relationship between total activity and the residual activity after a 2-h heat inactivation at 50 °C, for the 321 unique EK_L_ variants obtained from retest assays. Error bars of variant V15Q/R82P/C112S are standard deviations from twelve repeat samples.

The relationship between total activity and the stability as measured from residual activity after a two-hour heat challenge at 50 °C, is shown in Fig. 5 for the variants expressed at both 30 °C and 37 °C. The distributions for the variants expressed at the two different temperatures were very similar, and reveal that increased total activities were biased towards variants that were also more stable. Directed evolution studies often reveal a trade-off whereby variants with greater catalytic efficiency (eg. *k*_cat_) lead to some loss in stability. As a result, a minimum threshold stability is often required to accommodate mutations that improve activity^40,41^. Our assay for total activity represents an overall "fitness", which could improve via increases in several possible factors in addition to catalytic efficiency, including expression level, correct folding, solubility, and the stability of the protein. The non-linear correlation in Fig. 5 suggested that a stability threshold of approximately > 80% residual activity had to be reached before any mutations translated into an impact on total activity, regardless of which of the other factors the mutations were improving. The parent variant V15Q/R82P/C112S was below that threshold and so while selecting for total activity, the initial mutations were being selected indirectly for their stabilising impact.

### Statistical ranking of mutational effects

A partial least squares (PLS) analysis was used to deconvolute the effects of individual mutations on EK_L_ total activity and stability, from within the library of 321 unique EK_L_ variants. The 321 unique EK_L_ variants contained 206 unique nucleobase mutations, which included those found on the T7 promoter, *lac* operator, and ribosomal binding site as well as those encoding residues within the pelB secretion peptide and EK_L_ protein. Redundancies were removed from multiple mutations that always coexisted, by labelling as a single factor with multiple mutations (eg. I135S/t556g), which left a total of 170 mutations in 321 sequences. The frequency of each mutation within the 321 unique sequences, ranged from 1 to 93.

The PLS regression ranked the relative effects of all mutations (as independent variables) on the four dependent (response) variables of total activity and residual activity, each when expressed at 30 °C and at 37 °C. A tenfold cross validation resulted in coefficients for each mutation that were obtained from an optimal model (minimum root-mean PRESS) in which 7 components explained 63%, 65%, 70%, and 69% of the variance in activity 30 °C, activity 37 °C, stability 30 °C, and stability 37 °C, respectively, reflected in the Pearson correlations between predicted and actual responses of R^2^ = 0.63–0.7 (Supplementary Fig. S5).

The highest coefficient values indicate mutations that have the largest effects on each response in the model. High VIP values indicate mutations which account for more of the overall variability in the sequences themselves, and are therefore of more importance to the model. Mutations were first ranked by their variable importance in projection (VIP) values, to reject those with VIP < 0.6. The remainder were binned into significant mutations with VIP > 1.0, and moderately significant mutations with VIP 0.6 to 1.0, and their coefficients plotted in VIP rank order in Supplementary Fig. S5 for each of the four responses.

Silent mutations throughout the promoter, *lac* operator, ribosome binding site, pelB leader and EK_L_ gene, generally had insignificant impacts on any of the four responses. One exception is the silent mutation t556g in the EK_L_ gene, which potentially had an impact, but its effect cannot be isolated as this mutation was convoluted with I135S because all sequences with one of the mutations also always contained the other. One silent mutation, g226a (EK_L_ gene), had a significant negative impact on all four responses.

At the protein mutation level, S38T, I135S/t556g, A129T, S127T, M96L, and M100K were beneficial to both activity and stability. H235N was beneficial to activity, but neutral to stability. Then possibly I135V/T, M100T, Q160L, and M48K were also beneficial, but these mutations had VIP values of 0.84 to 0.95 making them less important to the PLS model. The pelB mutation P5’L had a positive impact but only on the total activity obtained when expressed at 37 °C. Notable mutations with significant to modest negative impacts were D142F, g226a, P162S, I128V, R124G, L74F, c604g and possibly G27S.

Interestingly, the ranking of mutation I135S, and to a lesser extent I135V or I135T, as highly beneficial for both total activity and stability, was consistent with a previous finding^26^ that mutation I135K improved the in vitro refolding of EK_L_. That patent also highlighted M48K as less significant, consistent with our ranking of this mutation as only moderately beneficial for total activity and stability. This validates our statistical analysis to be useful for differentiating between beneficial and deleterious mutations of EK_L_, including their relative impacts, and has the potential to inform further library designs or rational mutations to improve the properties of EK_L_.

### Purified EK_L_ variant characterization

Several variants were purified and then characterised in more detail to identify the broader links between their stability (*T*_m_ and kinetic inactivation rates), soluble expression level, enzyme kinetic parameters, and the total activity obtained in lysates. A total of 10 EK_L_ variants (V15Q, V15Q/R82P/C112S, WLEK0294, WLEK0362, WLEK0488, WLEK0513, WLEK0528, WLEK0699, WLEK0707, and WLEK0779), were expressed at both 30 °C and 37 °C. To examine the impact of codon-optimization, five variants were additionally codon-optimized to give V15Q_opt_, V15Q/R82P/C112S_opt_, WLEK0362_opt_, WLEK0513_opt_, and WLEK0528_opt_. WLEK0779, WLEK0528 and WLEK0528_opt_ could not be purified in sufficient amounts for characterisation. The residual activity after heat-inactivation in lysates indicated that WLEK0779 was not very stable, and that WLEK0528 was only moderately stable, and so this instability may have hampered their purification. EK_L_ purification used NTA-His_6_-tag affinity purification followed by affinity capture specific to the EK_L_ active-site using STI-immobilized agarose resin. While the first step would effectively capture all soluble EK_L_ proteins from clarified lysates, the second step would presumably bind only to correctly folded EK_L_ proteins with fully formed active-sites. The characterization results of those EK_L_ variants purified with sufficient amounts of protein, is summarised in Tables 2 and 3.

**Table 2:** Characterisation of EK_L_ variants expressed at 30 °C.

Variants	Total activity (lysate) (mM hr^−1^ L^−1^)		Specific activity (purified) (mM hr^−1^ ng^−1^ EK_L_)		Soluble expression (µg EK_L_ L^−1^)		*K*_m_ (mM)		*k*_cat_ (s^−1^)		*k*_cat_/*K*_m_ (mM^−1^ s^−1^)		*T*_m_ (°C)		*k*_u_ @ 45 °C (min^−1^)	
	AVG	SE	AVG	SE	AVG	SE	AVG	SE	AVG	SE	AVG	SE	AVG	SE	AVG	SE
WT CNY	28	12	n.d	–	n.d	–	n.d	–	n.d	–	n.d	–	n.d	–	n.d	–
V15Q	240	10	n.d	–	n.d	–	n.d	–	n.d	–	n.d	–	n.d	–	n.d	–
V15Q/R82P/C112S	4766	117	0.014	1.3E−03	341	30	0.58	0.08	247	23	425	73	48.0	0.04	0.0085	5E−04
WLEK0294	12,067	167	0.021	1.2E−03	567	32	0.58	0.13	321	43	553	141	48.7	0.05	0.0064	7E−05
WLEK0362	8725	40	0.020	3.4E−04	443	7	0.91	0.38	421	133	463	242	52.8	0.07	0.0017	5E−05
WLEK0488	14,081	2	0.010	3.3E−04	1365	43	0.26	0.06	81	10	309	82	49.2	0.05	0.0051	6E−05
WLEK0513	8462	52	0.015	3.4E−04	566	12	0.43	0.05	177	12	414	57	51.2	0.05	0.0025	1E−05
WLEK0528	8312	71	n.d	–	n.d	–	n.d	–	n.d	–	n.d	–	n.d	–	n.d	–
WLEK0699	17,634	196	0.025	6.4E−04	696	16	0.12	0.01	102	5	871	110	50.2	0.05	0.0043	1E−04
WLEK0707	7501	209	0.006	3.0E−04	1174	45	0.14	0.01	30	1	215	14	n.d	–	n.d	–
WLEK0779	19,081	197	n.d	–	n.d	–	n.d	–	n.d	–	n.d	–	n.d	–	n.d	–
V15Q_opt_	239	5	n.d	–	n.d	–	n.d	–	n.d	–	n.d	–	n.d	–	n.d	–
V15Q/R82P/C112S_opt_	6948	108	0.008	7.3E−04	841	73	0.25	0.06	65	8	261	71	48.1	0.05	0.0074	2E−04
WLEK0362_opt_	12,002	180	0.013	4.9E−04	939	33	0.23	0.04	93	7	411	77	52.7	0.07	0.0018	4E−05
WLEK0513_opt_	12,896	114	0.023	6.1E−04	567	14	0.42	0.04	253	15	606	72	51.2	0.09	0.0025	2E−05
WLEK0528_opt_	5965	73	n.d	–	n.d	–	n.d	–	n.d	–	n.d	–	n.d	–	n.d	–

Total activity in clarified lysates and specific activity for purified enzyme were each assayed using 0.0625 mM substrate. Soluble expression = Total activity/Specific activity. n.d.—no data.

**Table 3:** Characterisation of EK_L_ variants expressed at 37 °C.

Variants	Total activity (lysate) (mM hr^−1^ L^−1^)		Specific activity (purified) (mM hr^−1^ ng^−1^ EK_L_)		Soluble expression (µg EK_L_ L^−1^)		*K*_m_ (mM)		*k*_cat_ (s^−1^)		*k*_cat_/*K*_m_ (mM^−1^ s^−1^)		*T*_m_ (°C)		*k*_u_ @ 45 °C (min^−1^)	
	AVG	SE	AVG	SE	AVG	SE	AVG	SE	AVG	SE	AVG	SE	AVG	SE	AVG	SE
WT CNY	1*	15	n.d	–	n.d	–	n.d	–	n.d	–	n.d	–	n.d	–	n.d	–
V15Q	36	13	n.d	–	n.d	–	n.d	–	n.d	–	n.d	–	n.d	–	n.d	–
V15Q/R82P/C112S	8372	133	0.012	3.9E−04	698	20	0.28	0.04	74	6	260	42	47.9	0.10	0.0082	2E−04
WLEK0294	10,001	103	0.011	4.7E−04	894	37	0.21	0.03	67	5	316	57	48.4	0.07	0.0076	4E−04
WLEK0362	11,330	154	0.008	5.0E−04	1352	78	0.24	0.05	58	6	245	58	52.1	0.05	0.0021	7E−05
WLEK0488	9080	35	0.011	1.3E−04	847	10	0.25	0.06	87	9	354	92	48.8	0.05	0.0058	2E−04
WLEK0513	10,857	99	0.008	2.7E−04	1426	49	0.23	0.05	51	6	219	53	50.8	0.05	0.0032	3E−05
WLEK0528	1891	23	n.d	–	n.d	–	n.d	–	n.d	–	n.d	–	n.d	–	n.d	–
WLEK0699	10,107	82	0.041	4.7E−04	249	2	0.16	0.01	211	7	1329	104	49.1	0.2	n.d	–
WLEK0707	5619	47	n.d	–	n.d	–	n.d	–	n.d	–	n.d	–	n.d	–	n.d	–
WLEK0779	10,633	191	n.d	–	n.d	–	n.d	–	n.d	–	n.d	–	n.d	–	n.d	–
V15Q_opt_	62	11	n.d	–	n.d	–	n.d	–	n.d	–	n.d	–	n.d	–	n.d	–
V15Q/R82P/C112S_opt_	4645	67	0.019	7.0E−04	243	8	0.40	0.05	226	13	560	82	49.0	0.1	n.d	–
WLEK0362_opt_	4264	75	0.012	4.5E−04	343	11	0.52	0.04	168	8	324	30	52.7	n/a	n.d	–
WLEK0513_opt_	4382	20	0.013	1.3E−04	329	3	0.59	0.07	204	15	347	47	52.6	n/a	n.d	–
WLEK0528_opt_	3245	48	n.d	–	n.d	–	n.d	–	n.d	–	n.d	–	n.d	–	n.d	–

Total activity in clarified lysates and specific activity for purified enzyme were each assayed using 0.0625 mM substrate. Soluble expression = Total activity/Specific activity. n.d.—no data. * WT activity is rounded up to 1, but the limit of detection was approx 15 mM hr^−1^ L^−1^. igures.

### Enzyme kinetics for purified variants

To determine the kinetic parameters of each variant, initial velocity data were fitted by non-linear regression to a modified Michaelis–Menten equation, to account for any substrate inhibition which was apparent in all variants except the V15Q/R82P/C112S_opt_ expressed at 37 °C. All *K*_m_ values obtained were > 10 × the substrate concentration (0.0625 mM) used in the total activity assays and library screening, and hence *k*_cat_/*K*_m_ was the expected rate constant under these conditions. Furthermore, all substrate inhibition constants were found to be in the range 0.6–1 mM, which was thus an insignificant factor in the total activity assays and library screening performed at 0.0625 mM substrate.

**Figure 6:**
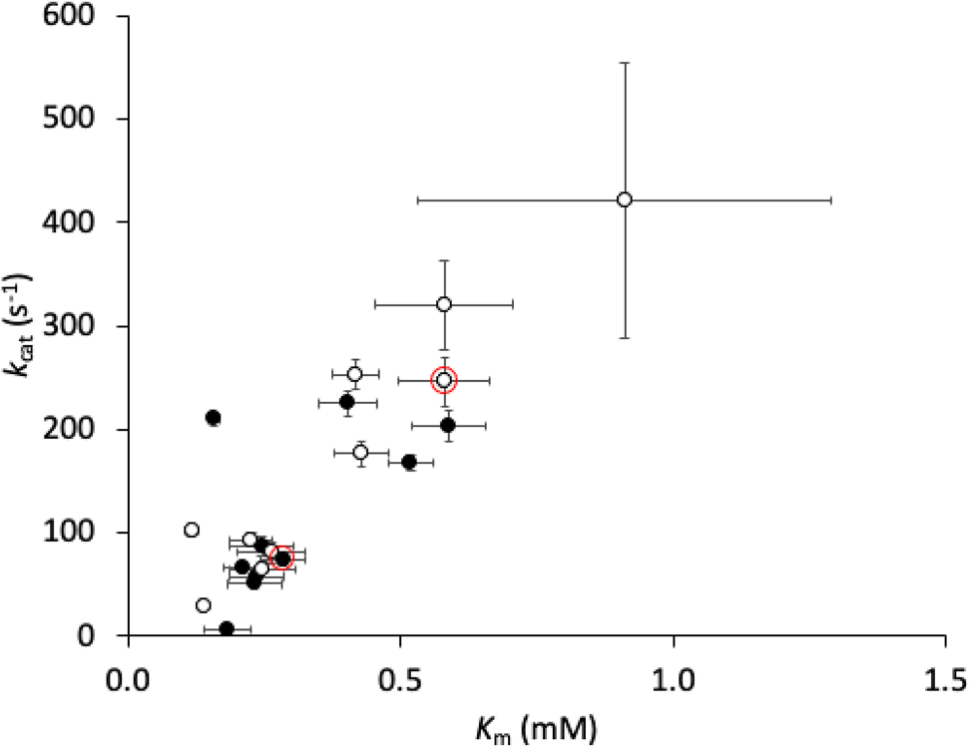
Relationship between catalytic turnover rate (*k*_cat_) and Michaelis–Menten dissociation constant (*K*_m_) for characterized variants expressed at (White circle) 30 °C and (Black circle) 37 °C. Red circles highlight the parent V15Q/R82P/C112S.

The relationship between the catalytic turnover rate constant (*k*_cat_) and substrate binding affinity (*K*_m_) for all EK_L_ variants, expressed at both 30 °C and 37 °C, is shown in Fig. 6. For most of the originally selected non-codon optimised variants, this revealed only minor differences in *k*_cat_ and *K*_m_, which mostly maintained *k*_cat_/*K*_m_ values within a narrow range 215–553 mM^−1^ s^−1^ and with an average standard error of ± 120 mM^−1^ s^−1^ (Tables 2 and 3). One exception was WLEK0699 which increased *k*_cat_/*K*_m_ up to fivefold relative to V15Q/R82P/C112S. Therefore, most of the variants were not altered in their catalytic efficiency, and so the total activity must have increased due to a change in level of soluble functional protein expression and its stability.

Changing the expression temperature and codon optimisation affected *k*_cat_ values, and to a lesser extent *K*_m_, indicating an impact on the proportion of the purified protein that was fully functional. Codon optimisation at 37 °C expression led to a threefold increase in *k*_cat_, but conversely a 50% decrease when expressed at 30 °C. Decreasing the expression temperature from 37 to 30 °C increased *k*_cat_ fourfold for non-codon optimised variants, but decreased it 30% for codon optimised variants. Thus overall, codon optimisation was preferable at the higher expression temperature, whereas non-codon optimisation was preferred at the lower temperature. On balance, the best combination for the highest *k*_cat_ was achieved with non-codon optimised variants expressed at 30 °C. This suggests that translation efficiency at the codon level needs to be matched by some other temperature-dependent aspect of translation, translocation, or folding within the protein expression pathway.

**Figure 7:**
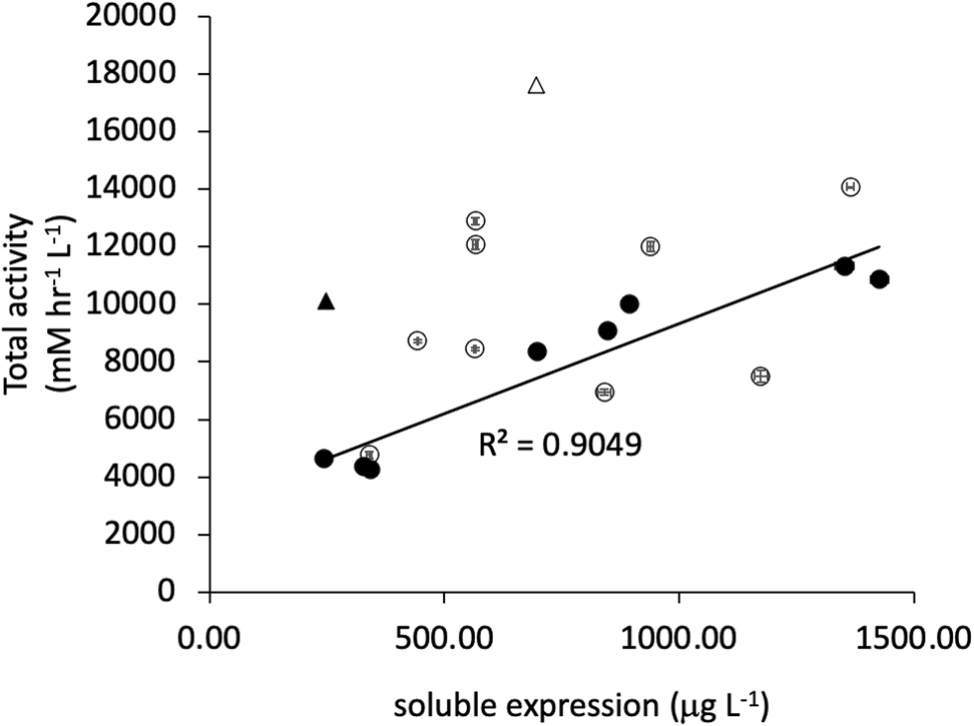
Relationship between soluble expression and total activity in clarified lysates. Variants were expressed at (White Circle) 30 °C or (Black Circle) 37 °C. WLEK0699 is shown separately as triangles from expression at (White Triangle) 30 °C or (Black Triangle) 37 °C.

It was expected that total activity would increase as a function of soluble protein expression level, especially as the specificity constants (*k*_cat_/*K*_m_) for purified variants did not vary significantly. The relationship between soluble protein expression and total activity is shown in Fig. 7 for variants expressed at 30 °C and 37 °C. WLEK0699, the only variant that increased *k*_cat_/*K*_m_, is plotted separately in each case (triangles). Soluble expression was determined by dividing the total activity in clarified lysates by the specific activity determined for the purified variants at the same substrate concentration of 0.0625 mM. Soluble expression was independently corroborated by SDS-PAGE densitometry, which was less accurate but confirmed the range of values obtained (Supplementary Fig. S6). As expected there was an underlying proportionality between soluble expression and total activity, although only strongly correlated at 37 °C (R^2^ = 0.9). The impact of increased *k*_cat_/*K*_m_ for WLEK0699 can be seen as an increase in total activity above the trend followed by the other variants.

### Thermostability of purified variants

Comparisons of enzyme kinetic parameters indicated that the proportion of correctly-folded soluble enzyme, was affected by expression temperature and codon optimisation. The thermostability of the purified variants was measured by differential scanning fluorimetry to probe folding quality further, as a possible source for differences in total activity, residual activity, and enzyme kinetics, when expressed under different conditions. The melting temperatures, *T*_m_, for purified variants are summarised in Tables 2 and 3. These *T*_m_ values were also observed to be inversely related to their unfolding rate constants at 45 °C providing an orthogonal verification of variant stability (Supplementary Fig. S7).

A comparison of *T*_m_ values to the residual activity after heat inactivation for 2 h at 50 °C, showed that variants with *T*_m_ < 48.4 °C, just below the incubation temperature, had a sharp decrease in residual activity from > 80% to < 10% (Supplementary Fig. S8), confirming that denaturation of the protein was the principle reason for loss of residual activity in the screening assays. The improvement in total activity for variants with > 80% residual activity (Fig. 5), therefore also translated into a threshold of *T*_m_ ≥ 48.4 °C required to achieve good expression and subsequently increased total activity for which the variants were selected (Supplementary Fig. S8B). Therefore, the *T*_m_ ≥ 48.4 °C threshold represents the minimal stability required to avoid unfolding, ensure periplasmic refolding in the periplasm, or provide resistance to proteolytic digestion or aggregation during expression at 37 °C.

The *T*_m_ values did not correlate with soluble expression levels, *k*_cat_, *K*_m_ or *k*_cat_/*K*_m_ when considering the entire dataset together, or after removing the variants with low stability (*T*_m_ < 48.4 °C). However, codon optimisation led to a noticeable 0.6–1.8 °C increase in *T*_m_ when expressed at 37 °C, yet no change when expressed at 30 °C. Meanwhile, decreasing the expression temperature from 37 to 30 °C slightly increased *T*_m_ by 0.4–1.1 °C for non-codon optimised variants, but decreased it by 0.8 °C for codon optimised variants. This directly mirrored the influence of these same expression condition factors on *k*_cat_ as discussed above, and linked an increase in *T*_m_ with an increase in *k*_cat_. Presumably both resulted from an improved proportion of the purified protein being in the functional native form, rather than being misfolded or aggregated at the time of measurement. Indeed it is often found that higher expression rates are detrimental to the soluble expression of aggregation-prone proteins^42^.

### Sequence and structural location for significant mutations

**Figure 8:**
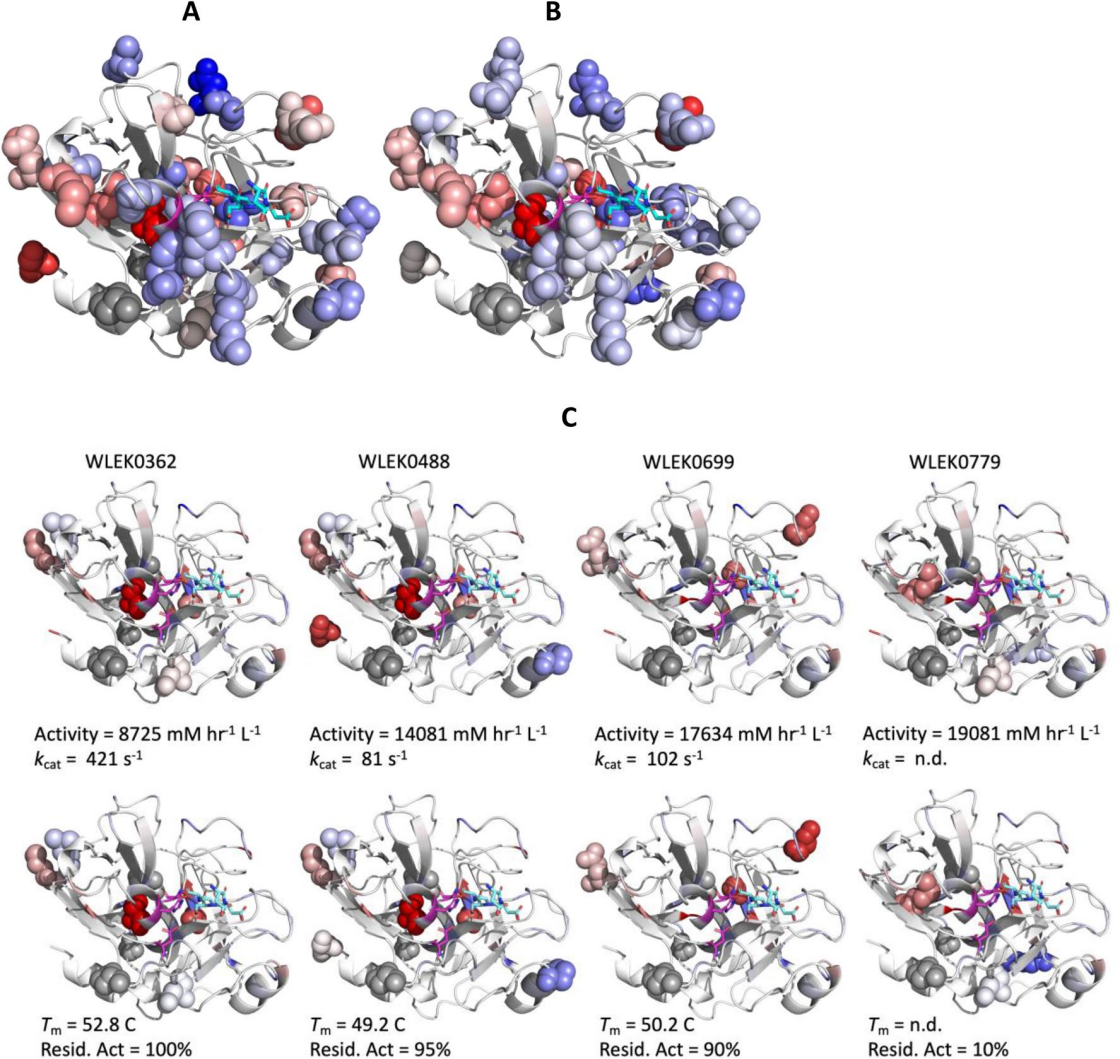
Position in EK_L_ structure of mutations with significant contributions to (**A**) Total activity, (**B**) Residual activity after heat inactivation, and (**C**) Key variants. Colours represent significant PLS coefficients from beneficial (red, 1.5) to deleterious (blue, -1.5) mutations. Sites with no observed mutation or with low PLS coefficients are shown in white. Grey spheres correspond to the positions of mutations in the parent sequence (V15Q, R82P, C112S) used as the backbone for DNA-shuffling. Residues in magenta are the catalytic triad (S187, H41, D92), while those residues in cyan show the DDDK peptide substrate. In Panel C, mutation sites for each variant are highlighted as spheres, and the colours represent PLS coefficients for total activity (top row) and residual activity after heat inactivation (bottom row). Structure was generated from PDBID: 1EKB.

The 321 unique variants used in the PLS model represented a wide range of activities and stabilities obtained from the libraries, and not just those selected for improvements. The locations and impact of all mutated sites in the protein are highlighted by sequence position and secondary structure type in Supplementary Fig. S5I, while those with highly positive (beneficial) or highly negative (detrimental) PLS coefficients are highlighted in the structure of EK_L_ in Fig. 8, along with the locations of mutations present in key variants. The locations of all mutations were highly distributed throughout the sequence, with the C-terminal helix being the only secondary structure element with no mutations observed. Analysis by sequence and secondary structure alone revealed no overall bias in amino acid substitution types or their location, for either beneficial or detrimental mutations, with both types simply reflecting the overall amino-acid and secondary structure content of the protein. However, some general observations were found when considering certain substitution types in the context of structure.

As might be expected from the increase in backbone flexibility they can cause, mutations away from proline were detrimental (P162S, P214L), while the only switch to proline was the stabilising consensus mutation R82P. Similarly, mutations towards glycine can increase backbone flexibility, and this may explain the destabilising effect of R124G, although this mutation also removed a salt bridge.

Mutations that broke or formed salt bridges featured highly. Several mutations at charged residues were detrimental (D21N, R124G, D142F, K156R). Except for D142F, These would have all broken salt bridges (D21-K52; R124-E195; K156-D176). Mutations towards charged residues were beneficial (M100K and M48K), although Q137R was beneficial for activity but deleterious to stability. M100K has the potential to produce a new salt bridge to the unpaired residue E99, while M48K has the potential to form a new salt bridge with neighbouring residue E49. Q137 is close to two (9.5 Å and 10 Å) of the aspartate sidechains of the DDDK substrate when bound, and so the mutation to arginine potentially improved substrate binding through increased electrostatic interactions.

Finally, mutations at charged residues not already in salt bridges were also beneficial (E99A, H235N). E99 is a partially buried negative charge which is thermodynamically unfavourable and so the E99A mutation would favourably remove that charge. Alternatively, as discussed above E99 can potentially form a salt bridge in variants with the beneficial mutation M100K. H235 is at the C-terminus and so the impact of H235N in this flexible and solvent exposed region is harder to rationalise.

One minor structural motif was apparent in the beta-strand at residues G123 to G130, with a binary repeat pattern in the sequence. This resulted in positive mutations appearing on one face of the strand and negative mutations on the other, while avoiding mutation of C126 which forms a disulphide bond to C193. Thus improved contacts could be achieved on the face that interacts with the N-terminal loop region (residues D6 to W14), and also the neighbouring strands within the same beta-sheet.

While the mutations with greatest importance (VIP > 0.5) were distributed throughout the structure, the positive or negative PLS coefficients for both total activity and stability were segregated such that mutations with negative coefficients were generally closer to catalytic residues or the substrate. This strongly indicates that mutations most detrimental to total activity were those that impacted structural regions directly involved in catalysis or substrate binding. By contrast, those that improved total activity mostly acted indirectly at a distance from the active site, through improved soluble expression and stability. Thus the best mutations for improved total activity tended to be found at a distance from the active site. This is in contrast to most directed evolution studies which have started with good expression and so focussed on improving catalytic efficiency (eg. *k*_cat_). In those cases, beneficial mutations are more often found closer to the active site^43^. In our study, it appears that the biggest gains in total activity were easier to obtain through improved stability and expression, which started from a low point, than from improvement of catalytic efficiency.

Most of the observed mutations can be rationalised post hoc through the local interactions gained or lost, or through altered backbone flexibility expected for mutations to and from proline (eg. P162S and P214L), as described above. The individual impacts of some of these important mutations are described below in the context of key selected variants.

### Comparison of selected variants

Variant WLEK0779 (V15Q/R82P/M96L/C112S/R124G/N169D) exhibited the highest total activity when expressed at 30 °C, and yet only moderate total activity when expressed at 37 °C, and just 11% residual activity after pre-incubation at 50 °C for 2 h. Examination of Fig. 8C shows how this high activity and yet poor stability resulted from a balance of beneficial and deleterious mutations. M96L was beneficial to both activity (coeffs. 0.71 at 30 °C; 0.76 at 37 °C), and stability (coeffs. 0.68 at 30 °C; 0.56 at 37 °C). However, R124G was slightly deleterious to activity (coeffs. − 0.24 at 30 °C; − 0.36 at 37 °C), but highly deleterious to stability (coeffs. − 0.92 at 30 °C; − 0.75 at 37 °C). N169D was close to neutral for stability, with a slight benefit to activity (coeffs. 0.2 at 30 °C; 0.1 at 37 °C). Thus, carrying the R124G mutation was the likely cause of rapid inactivation of WLEK0779 during the 2 h incubation at 50 °C, and lower total activity when expressed at 37 °C. The R124G mutation removes a salt bridge to E195, while introduction of a glycine would also potentially increase the local backbone flexibility. Indeed, variant WLEK0463 (V15Q/R82P/M96L/C112S/N169D) was identical except for the R124G mutation, and exhibited considerably better stability with 78% and 92% residual activity from 30 and 37 °C expression respectively. However, its total activity, while twofold improved over V15Q/R82P/C112S, was only 30–40% that of WLEK0779.

WLEK0362 (V15Q/S38T/L74F/R82P/M100K/C112S/S127T/N169D) was the most stable variant with 93–100% retained activity, and the highest *T*_m_ of all when purified. It also had 1.3 to 1.8-fold improved total activity over V15Q/R82P/C112S. Examination of Fig. 8C shows that the high stability and good activity resulted from mutations that were mainly beneficial, and particularly so for stability. S38T had the highest positive PLS coefficients for both activity (coeffs. 1.5 at 30 °C; 1.6 at 37 °C), and stability (coeffs. 2.0 at 30 °C; 1.7 at 37 °C), and had the highest importance to the model (VIP of 6.35). S127T was also strongly positive to activity (coeffs. 0.66 at 30 °C; 0.67 at 37 °C), and stability (coeffs. 1.0 at 30 °C; 0.83 at 37 °C), and M100K slightly less positive (coeffs. in the range 0.35 to 0.57). As described above N169D was essentially neutral to all responses. Finally the effects of L74F were only modestly detrimental (coeffs. in the range − 0.16 to − 0.27) even though its VIP of 4.7 indicates that it was important to the model.

WLEK0488 (V15Q/S38T/L74F/R82P/M100K/C112S/S127T/P162S/H235N) had slightly lower stability, higher total activity in lysates, and fivefold lower *k*_cat_ than WLEK0362. The biggest difference was due to P162S which was deleterious to both activity (coeffs. − 0.55 at 30 °C; − 0.58 at 37 °C), and stability (coeffs. − 0.66 at 30 °C; − 0.54 at 37 °C). This was counteracted by H235N for activity (coeffs. 1.1 at 30 °C; 0.77 at 37 °C), but not for stability (coeffs. 0.08 at 30 °C; 0.06 at 37 °C).

Finally, WLEK0699 (P5'L/A19'V/V15Q/R82P/E99A/C112S/A129T/I135S) had improved stability (84–89% retained activity) and a high total activity (5 to sixfold improved over V15Q/R82P/C112S). As discussed above, this variant was the only one which increased catalytic effciency, such that *k*_cat_/*K*_m_ increased up to fivefold relative to V15Q/R82P/C112S, compared to the other variants which improved total activity through increased soluble expression. From Fig. 8C it can be seen that this variant introduced three beneficial mutations, E99A, A129T and I135S, and none that were detrimental. A129T was strongly positive to activity (coeffs. 0.79 at 30 °C; 0.78 at 37 °C), and stability (coeffs. 1.0 at 30 °C; 0.81 at 37 °C). I135S was even more positive to activity (coeffs. 0.89 at 30 °C; 1.03 at 37 °C), and stability (coeffs. 1.2 at 30 °C; 0.91 at 37 °C). E99A was modestly beneficial to activity (coeffs. 0.32 at 30 °C and 37 °C), and stability (coeffs. 0.34 at 30 °C; 0.25 at 37 °C). The promoter region mutations P5'L and A19'V were much less influential with either low coefficients (P5'L) or low VIP (A19'V).

The improved catalytic efficiency of WLEK0699 was driven mainly through a decrease in *K*_m_ observed at either expression temperature, and an increase in *k*_cat_ seen only when expressed at 37 °C. The A129T and I135S mutations were both found near the EK_L_ extended binding site, and hence potentially influencing *k*_cat_ or *K*_m_. The A129T alanine sidechain was 7.0 Å from the backbone amide nitrogen of catalytic residue S187, and so the threonine mutation gives it the potential to hydrogen bond directly to the carbonyl of neighbouring residue G188 to create a more stable active site. The A129 sidechain also packs onto the V15 sidechain in wildtype EK_L_, which had been mutated to V15Q in the parent sequence. Thus the benefits of A129T may also have been complementary to the V15Q mutation. The I135 mainchain was 10 Å from the lysine sidechain of the substrate DDDK, although separated by the disulphide formed between C183 and C211. The I135 sidechain is also highly solvent exposed and in the middle of a hydrophobic patch with V2, L134, Y136, and A212, making a I135S mutation potentially beneficial to solubility.

The E99 sidechain makes van der Waals contacts with the hydrophobic M100 and L74 sidechains, which unfavourably partially buries an acidic sidechain charge. E99A would remove this unfavourable charge. It is worth noting that M100K which has positive PLS coefficients as described above, would potentially be stabilising by forming a new salt bridge to E99. E99A and M100K only appear together once (in WLEK0433), out of 72 instances of variants containing M100K and 24 instances containing E99A, highlighting a strong selection against the two simultaneous mutations. WLEK0433 ranked low, with little improvement over the V15Q/R82P/C112S parent.

## Conclusions

Cytoplasmic expression of bovine enterokinase light chain (EK_L_) in *E. coli* leads to inclusion body formation and no active soluble enzyme. Current production therefore requires solubilisation and refolding. Periplasmic expression using a pelB fusion enabled a very low level of native enzyme activity to be recovered directly when expressed at 30 °C in *E. coli* C41(DE3) and after concentrating the cells. An initial combinatorial scan of consensus mutations successfully improved the total activity of enterokinase enzyme activity detected in clarified lysates, by at least 340-fold. Adopting V15Q and V15Q/C112S as the initial parent variants, error-prone PCR enabled 225 variants with 1.2- to fivefold improvement in total activity (relative to V15Q) to be identified from a screen of 3000 colonies. Sequencing of these identified 86 mutations that were recombined through DNA-shuffling in four rounds, with a total of ~ 6500 colonies screened. This finally led to 321 unique variants from which the top variants had total activity in clarified lysates up to 680-fold higher for 30 °C expression, and up to 11,300-fold higher for 37 °C expression, compared to the wild-type chinese yellow enterokinase. While the catalytic efficiencies of the selected high-activity variants were mostly unchanged, many had become more stable as measured by residual activity after heating at 50 °C for 2 h. The melting temperatures for purified variants confirmed that exceeding a threshold level of > 80% residual activity, which equated to a *T*_m_ > 48.4 °C, was important to enable variants to evolve increased total activity when selected from expression at 37 °C. Thus increased stability was a major factor that enabled a greater overall yield of enzyme in the soluble and native form during expression, and hence higher total activities. One interesting observation was that the use of codon optimisation interacted with the expression temperature to affect the quality of the protein, where on average the highest *k*_cat_ and *T*_m_ of the enzyme variants were obtained when expressing non-codon optimised variants at 30 °C.

Statistical analysis by PLS allowed the importance and influence of each mutation within the unselected library to be ranked, and then mapped to the protein structure. Mutations detrimental to total activity and stability were clustered around the active site. Thus the overall best mutations for total activity, which is itself improved with increased stability, tended to be found at a distance from the active site. This is in contrast to most other studies where catalytic efficiency (eg. *k*_cat_) is the target for improvement of activity, for which beneficial mutations are most often found closer to the active site.

The final variants obtained now enable efficient expression of active enterokinase directly in *E. coli*, without the need for in vitro refolding. The expression levels of up to 1.5 mg L^−1^ in 400 mL shake flask cultures promises even greater yields obtainable by scale up and intensification to higher cell densities in bioreactors.

## Supplementary Information

Supplementary Information.

## Data Availability

The datasets used and analysed during the current study are available in the UCL Research Data Repository (https://doi.org/10.5522/04/20477127), and also from the corresponding author on reasonable request.
