# Supplementary material for: Directed evolution for soluble and active periplasmic expression of bovine enterokinase in Escherichia coli
Source: Sci Rep. 2022 Oct 21;12:17721. doi: 10.1038/s41598-022-22574-6 (PMC9587228; doi:10.1038/s41598-022-22574-6)
Supplement: Supplementary file 1 — Supplementary Information. [file 41598_2022_22574_MOESM1_ESM.docx]

# Supplementary Information for:

# Directed evolution for soluble and active periplasmic expression of bovine enterokinase in Escherichia coli

Weiluo Lee, Subhas Pradhan, Cheng Zhang, Niccolo Venanzi, Weina Li, Stephen Goldrick, Paul A. Dalby

**S1. EK_L_ sequence**

**A**


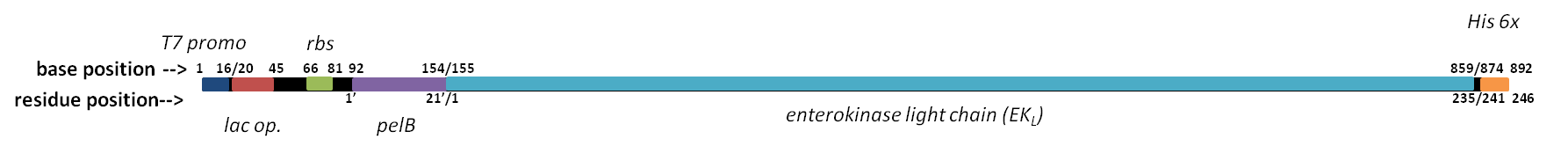


**B**

10 20 30 40 50
IVGGSDSREG AWPWVVALYF DDQQVCGASL VSRDWLVSAA HCVYGRNMEP
 60 70 80 90 100
SKWKAVLGLH MASNLTSPQI ETRLIDQIVI NRHYNKRRKN NDIAMMHLEM
 110 120 130 140 150
KVNYTDYIQP ICLPEENQVF PPGRICSIAG WGALIYQGST ADVLQEADVP
 160 170 180 190 200
LLSNEKCQQQ MPEYNITENM VCAGYDAGGV DSCQGDSGGP LMCQENNRWL
 210 220 230

LAGVTSFGYQ CALPNRPGVY ARVPRFTEWI QSFLH

**Figure S1. Sequence elements for EK_L_ expression, including numbering system for nucleobases and amino acid residues. A)** Sequence elements included a T7 promoter (T7 promo), lac operator (lac op), ribosomal binding site (rbs), pelB signal peptide (pelB), Cny bovine enterokinase light chain (EK_L_), and His6x affinity tag. For example, a variant with the following notation, P5’L/V15Q; t16a [T7 promo], g127t [pelB], c166t [EK_L_] indicates a Pro to Leu mutation at position 5 of the pelB sequence, and a Val to Gln mutation at position 15 of the EK_L_ sequence. The DNA level mutations are numbered contiguously, and the sequence elements in brackets indicate the location of each mutated base. **B)** Cny bovine EK_L_ protein sequence, with secondary structure highlighted as loop (green), helix (magenta), sheet (yellow).

**S2. Consensus sequence analysis**

A multiple alignment of 250 sequences was generated based on a BLAST (Ye et al 2006) search with Cny enterokinase light chain sequence as the query, and then aligned using ClustalW (Thompson et al 1994). Consensus sequences were generated (Figure 2) in PFAAT (Caffrey et al. 2007). Buried residues and proline residues in loop regions, also avoiding the active site, were considered for mutation to give the potential mutation sites shown in Table S1.

Caffrey DR, Dana, PH, Mathur V, Ocano M, Hong EJ, Wang YE, Somaroo S, Caffrey BE, Potluri S, Huang ES (2007) PFAAT version 2.0: a tool for editing, annotating, and analyzing multiple sequence alignments. *BMC. Bioinformatics.* 8:381.

Thompson JD, Higgins DG, Gibson TJ (1994) CLUSTAL W: improving the sensitivity of progressive multiple sequence alignment through sequence weighting, position-specific gap penalties and weight matrix choice. *Nucleic Acids Res* 22:4673–4680

Ye J, McGinnis S, Madden TL (2006) BLAST: improvements for better sequence analysis. *Nucleic Acids Res* 34:W6–W9

**
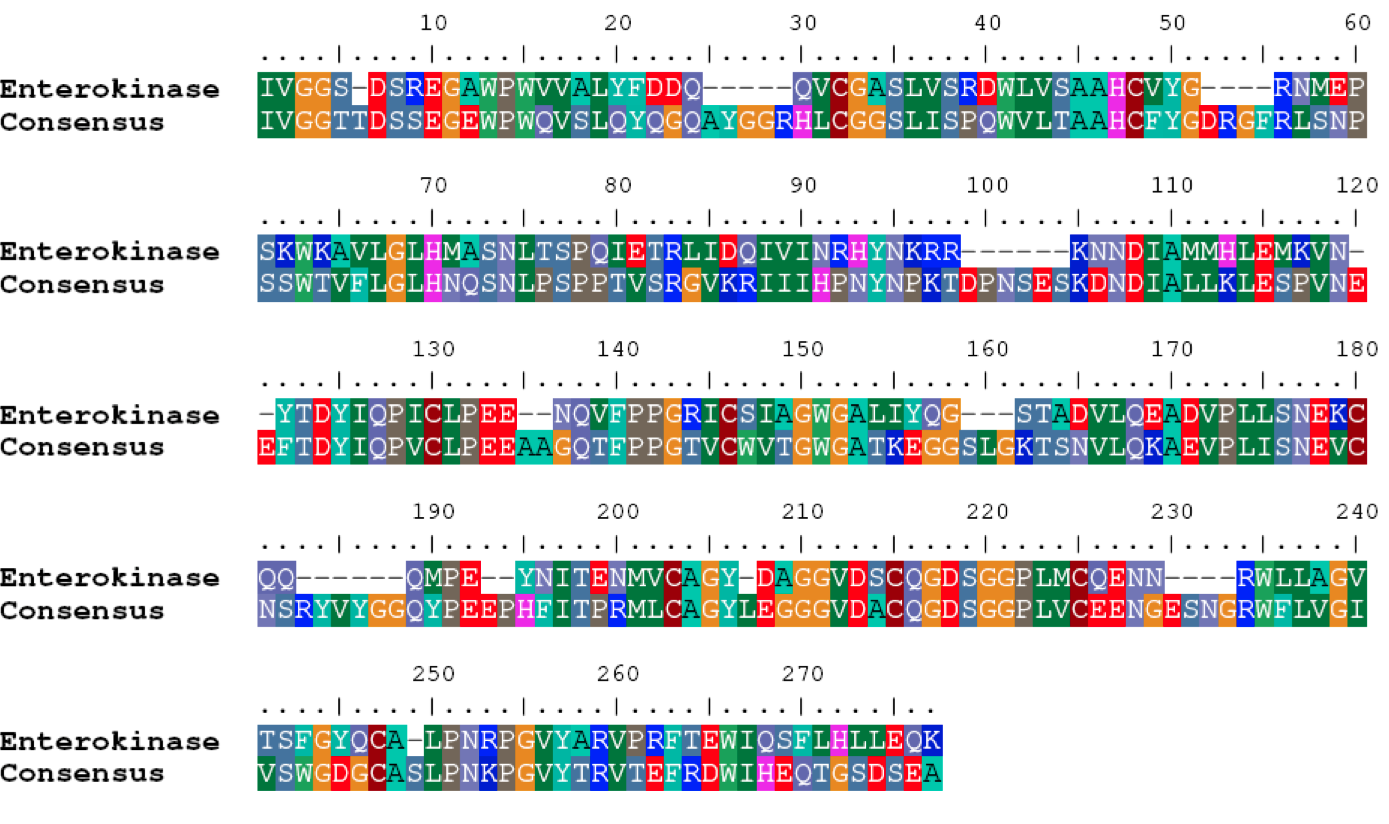
**

**Figure S2. Enterokinase sequence aligned to the consensus sequence.** Sequences were obtained via BLAST search. 250 sequences were used to generate a multiple sequence alignment in Clustal W, and a consensus sequence generated using PFAAT. Residues have been shaded depending on residue similarity to significant changes between the two sequences.

| Residue position | Cny EK residue | Consensus Mutation | Alignment numbering |
| --- | --- | --- | --- |
| 15 | V | Q | 16 |
| 17 | A | S | 18 |
| 28 | A | G | 29 |
| 33 | R | P | 39 |
| 36 | L | V | 42 |
| 37 | V | L | 43 |
| 38 | S | T | 44 |
| 43 | V | F | 49 |
| 55 | A | V | 65 |
| 69 | Q | P | 79 |
| 82 | R | P | 92 |
| 101 | K | P | 117 |
| 128 | I | V | 148 |
| 168 | E | P | 199 |
| 202 | A | V | 238 |

Table S1: Potential mutation sites identified from the consensus sequence. Alignment numbering is taken from Figure S2.

**S3. Initial consensus mutant analysis of expression and activity**

Cells expressing the pelB-fused Chinese northern yellow Cny-EK_L_ (UniProtKB Q6B4R4) EK­_L_ protein sequence (pelBcnyEK) or consensus mutant variants, were grown at 37 ^0^C to OD_600_ 0.8 in 400 mL LB with 40 μg/mL kanamycin in 2.5 L shake flasks, induced at OD_600_ of 0.8, with 0.15 mM IPTG and then grown at 30 ^0^C for 7 hours.

For the soluble fraction analysis, 200 mL of the cultures were centrifuged at 2400 x g, then resuspended in 20 mL 20 mM Tris, 5 mM EDTA and 30% sucrose at pH 8 to extract the periplasmic fraction by osmotic shock. The solution was incubated on ice for 20 minutes before centrifugation at 8500g for 25 minutes. The supernatant (sucrose fraction) was saved for analysis and the pellet resuspended in 20 mL of 5 mM MgCl_2_. This solution rapidly expands the cells via osmosis, rupturing the outer membrane and releasing periplasmic proteins. This solution was then incubated on ice for a further 15 minutes before a second centrifugation at 8500g for 25 minutes. The supernatant was collected (periplasmic fraction) and both the sucrose and periplasmic fractions were dialysed into reaction buffer (70 mM Tris, 2 mM CaCl_2_, 50 mM NaCl at pH 8) for analysis of EK activity. For the insoluble fraction analysis, 200 mL cell cultures were centrifuged at 2400g, resuspended in 10 mL 20 mM Tris, 0.5 M NaCl, 5 mM EDTA, pH 8, then lysed by sonication. Soluble and insoluble fractions were separated by centrifugation at 8000g for 30 minutes at 4 °C.

The total activity of each fraction was determined from cleavage of the GD4K-na fluorogenic substrate using a FLUOstar OPTIMA microwell plate spectrophotometer (BMG Labtech, UK), 340 nm excitation and 420 nm emission to monitor initial rates. Reactions were initiated by auto-injection of 10μL/well of 1.25 mM GD4K-na substrate pre-dissolved in the reaction buffer, giving final reaction conditions of 0.0625 mM GD4K-na, 70 mM Tris-HCl, 2 mM CaCl_2_, 50 mM NaCl, pH 8 at 25 °C.

SDS-PAGE densitometry was carried out with 12.5 % Tris-Glycine pre-cast gels (Invitrogen Ltd.). Protein samples were prepared by first mixing 50μL sample with 50μL 2x Laemli sample buffer and then heated to 99 °C for 15 minutes before cooling to room temperature. 5 μL of sample was loaded into each well with 5 μL of PageRuler Prestained Protein Ladder (Fermentas Life Sciences) as a marker. Additionally, 5μL/well of 0.125mg/mL BSA and 0.03125mg/mL BSA were loaded as reference protein standards for comparison purposes.

Gels were run with Tris-Glycine buffer (stored as 10x buffer: 144 g/L Glycine, 30 g/L Tris-base and 10 g/L SDS) at 100 V for 120 minutes. Gels were stained in 150 mL of Coomasie blue (50 % methanol, 10 % acetic acid and 0.5g/L brilliant blue) for 30-45 minutes. Gels were destained in 100mL of destaining reagent (40 % methanol and 10 % acetic acid) for 20-30 minutes and repeated as required. Gels were stored in pure water before being visualized using a Gel-Doc IT Imaging System (UVP Bioimaging Systems, Inc.). Densitometry analysis was carried out using Labworks V4.5 (UVP Inc., Media Cybernetics Inc.).

**A**


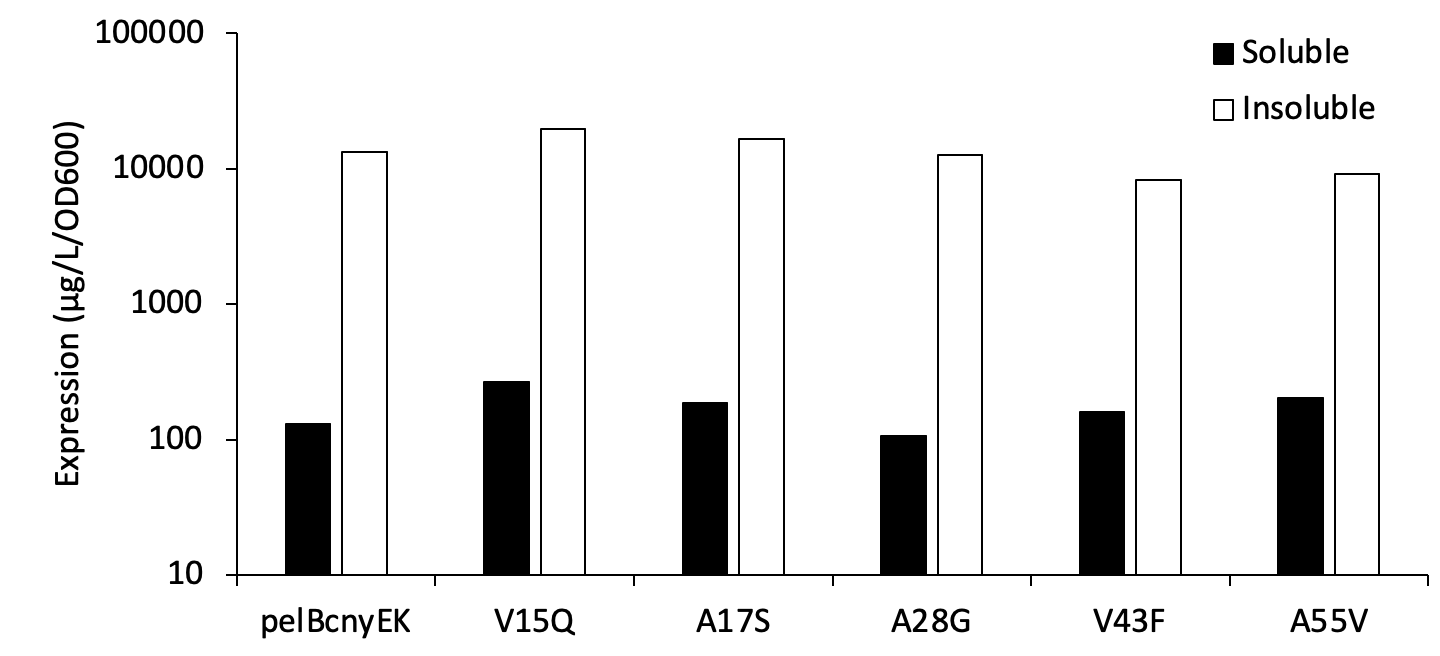


**B**


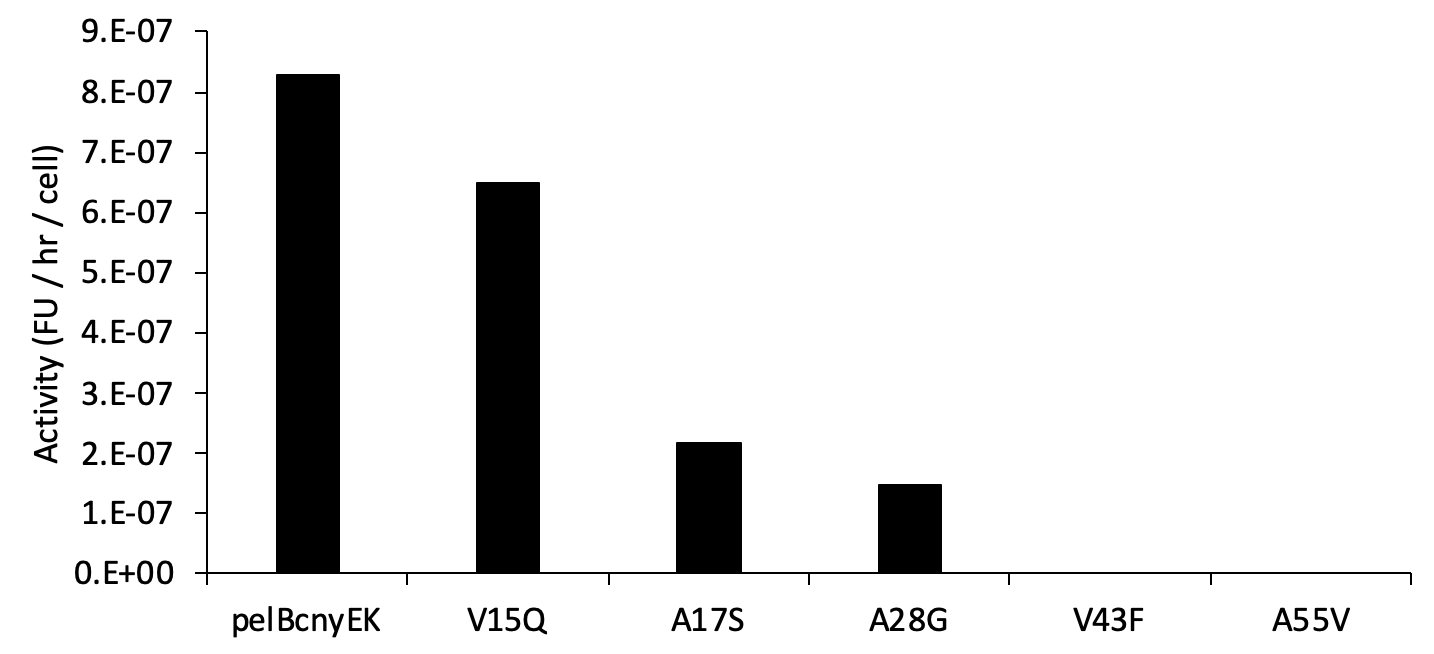


Figure S3. Soluble and insoluble expression levels (A), and total activity (B) of WT and rationally designed variants of cnyEK. Variants were expressed (n=1) in *E. coli* BL21 DE3 at 30 °C in shake flasks. Expression levels were determined by SDS-PAGE densitometry. Expression and total activity are normalised to the final cell OD600, while the total activity is converted to a per cell value using OD_600_ of 1 as equivalent to 10^9^ cells.

S4. Directed evolution libraries and analysis

Table S2 – Random mutations discovered and used in DNA-shuffling libraries

| **Library & Description** | **Mutation(s)** | **Gene** | **Primer Sequence (5’ – 3’)** |
| --- | --- | --- | --- |
| L1: mutations derived from bottom-tiered variants | A141V | EK_L_ | atctatcaaggaagtactgtggatgtgttacaggaggcg |
|  | E115D | EK_L_ | caaccaatttctctgccggatgaaaaccaggtcttccca |
|  | P162S | EK_L_ | aaatgtcagcaacagatgagcgaatataacattacggaa |
|  | R8H | EK_L_ | gtagggggctctgattcgcatgagggggcatggccgtgg |
| L2: mutations derived from bottom-tiered variants | A11T | EK_L_ | tctgattcgcgtgaggggacctggccgtggcaggtggct |
|  | I135V | EK_L_ | gccggctggggagcgcttgtgtatcaaggaagtactgcg |
|  | N91S | EK_L_ | aacaaacgccgtaaaaacagcgatattgccatgatgcac |
|  | Y175F | EK_L_ | aatatggtatgcgcagggtttgatgccggtggcgttgac |
| L3: mutations derived from middle-tiered variants | E99D | EK_L_ | attgccatgatgcacttagatatgaaagtgaactatacg |
|  | H235N | EK_L_ | tggattcagtctttcctcaacgcggccgcactcgagcac |
|  | I125V | EK_L_ | gtcttcccaccaggtcgcgtgtgttccatagccggctgg |
|  | I70V | EK_L_ | aatctgacgagcccgcaagtggagacgcgacttatcgac |
|  | M100T | EK_L_ | gccatgatgcacttagaaaccaaagtgaactatacggat |
| L4: mutations derived from top-tiered variants | G27D | EK_L_ | gatgatcaacaggtctgcgatgcctctttagtgtctcgt |
|  | M48K | EK_L_ | tgtgtgtacggccgtaacaaagaaccgtcaaaatggaag |
|  | M96L | EK_L_ | aacaatgatattgccatgctgcacttagaaatgaaagtg |
|  | N169D | EK_L_ | gaatataacattacggaagatatggtatgcgcagggtat |
|  | R124G | EK_L_ | caggtcttcccaccaggtggcatctgttccatagccggc |
| L5: mutations derived from top-tiered variants | K156R | EK_L_ | cccctgctgtcgaatgaacgttgtcagcaacagatgccc |
|  | L74F | EK_L_ | ccgcaaatcgagacgcgatttatcgaccaaatagtaatt |
|  | M100K | EK_L_ | gccatgatgcacttagaaaaaaaagtgaactatacggat |
|  | S127T | EK_L_ | ccaccaggtcgcatctgtaccatagccggctggggagcg |
|  | S38T | EK_L_ | tctcgtgattggctggtgaccgcagcgcactgtgtgtac |
| L6: expression or secretion-related mutations only | c9t | T7 promo | tcccgcgaaattaatacgattcactataggggaattgtg |
|  | L4’R | pelB | atacatatgaaatacctgcggccgaccgctgctgctggt |
|  | t41a | lac op | aattgtgagcggataacaaatcccctctagaaataattt |
| L7: expression or secretion-related mutations only | c125t | pelB (silent) | gaccgctgctgctggtctgttgctcctcgctgcccagcc |
|  | a12g | T7 promo | cgcgaaattaatacgactcgctataggggaattgtgagc |
|  | M20’I | pelB | tcgctgcccagccggcgatagccattgtagggggctctg |
|  | t42c | lac op | aattgtgagcggataacaatccccctctagaaataattt |
|  | t41c | lac op | aattgtgagcggataacaactcccctctagaaataattt |
| L8: expression or secretion-related mutations | c13a | T7 promo | gcgaaattaatacgactcaatataggggaattgtgagcg |
|  | a79g | rbs | ttgtttaactttaagaaggggatatacatatgaaatacc |
|  | Q17’H | pelB | tgctgctcctcgctgcccatccggcgatggccattgtag |
| L9: remaining mutations from lower, mid, and top-tiered variants | K54R | EK_L_ | atggaaccgtcaaaatggcgtgcagtactggggttgcac |
|  | K86R | EK_L_ | attaatccgcactataaccgtcgccgtaaaaacaatgat |
| L10: remaining mutations (from variants ≥ 1.2x improved over V15Q or V15Q/C112S) | A129T | EK_L_ | ggccgtatttgcagcattaccggctggggcgcgctga |
|  | A141T | EK_L_ | ggcagcaccaccgatgtgctg |
|  | A141T/D142F | EK_L_ | ggcagcaccacctttgtgctgcagga |
|  | P5’L | pelB | tacatatgaaatacctgctgctgaccgctgctgctggtc |
|  | D142F | EK_L_ | ggcagcaccgcgtttgtgctgcagga |
|  | D21N | EK_L_ | caggtggcgctgtattttaacgatcagcaggtgtgcggc |
|  | D21N/D22E | EK_L_ | caggtggcgctgtattttaacgaacagcaggtgtgcggcgcg |
|  | D22E | EK_L_ | gtggcgctgtattttgatgaacagcaggtgtgcggcgcg |
|  | G27S | EK_L_ | cagcaggtgtgcagcgcgagcctggt |
|  | I111V | EK_L_ | accgattatattcagccggtgagcctgccggaagaaaac |
|  | I128V | EK_L_ | ccgggccgtatttgcagcgtggcgggctggggcgcgctg |
|  | I128V/A129T | EK_L_ | ccgggccgtatttgcagcgtgaccggctggggcgcgctga |
|  | I135S | EK_L_ | ggctggggcgcgctgagctatcagggcagcacc |
|  | I135S/Q137R | EK_L_ | ggggcgcgctgagctatcgtggcagcacc |
|  | I135T | EK_L_ | ggctggggcgcgctgacctatcagggcagcacc |
|  | I135T/Q137R | EK_L_ | ggggcgcgctgacctatcgtggcagcacc |
|  | L191I | EK_L_ | ggcgatagcggcggcccgattatgtgccaggaaaacaac |
|  | L200M | EK_L_ | caggaaaacaaccgttggatgctggcgggcgtgaccagc |
|  | N165D | EK_L_ | cagcagatgccggaatatgatattaccgaaaacatggtg |
|  | P214L | EK_L_ | ggctatcagtgcgcgctgctgaaccgtccgggcgtgtat |
|  | Q137R | EK_L_ | tgatttatcgtggcagcacc |
|  | Q160L | EK_L_ | aacgaaaaatgccagcagctgatgccggaatataacatt |
|  | Q24L | EK_L_ | gatcagctggtgtgcggcgcgagcctg |
|  | Q24L/G27S | EK_L_ | gatcagctggtgtgcagcgcgagcctggtgagccgt |
|  | R8P | EK_L_ | gtgggcggcagcgatagcccggaaggcgcgtggccgtgg |
|  | S63N | EK_L_ | ctgggcctgcatatggcgaacaacctgaccagcccgcag |
|  | t25a, t16a | lac op, T7 promo | taatacgactcactaaaggggaatagtgagcggataacaattcc |
|  | V180A | EK_L_ | ggctatgatgcgggcggcgcggatagctgccagggcgat |
|  | Y84H | EK_L_ | attgtgattaacccgcatcataacaaacgtcgtaaaaac |
|  | t16a | T7 promo | taatacgactcactaaaggggaattgtgagcgg |

Table S3 – EK_L_ variants used for HITS-shuffling in libraries L11 and L12.

| **Library & Description** | **Variant ID** | **pelB-EK Protein Mutations;**  **DNA Mutations** |
| --- | --- | --- |
| L11: DNA-shuffling of top 2 variants from libraries L1 – L6 & L8 – L9 | WLEK0240 | V15Q/S38T/L74F/M100K/C112S/S127T |
|  | WLEK0245 | V15Q/I70V/M100T/C112S/I125V/H235N |
|  | WLEK0294 | V15Q/S38T/L74F/M100K/C112S/S127T/P162S |
|  | WLEK0306 | V15Q/S38T/L74F/C112S/A141V/P162S |
|  | WLEK0324 | V15Q/S38T/L74F/M100K/C112S/S127T |
|  | WLEK0335 | V15X/E99D/C112S/I125V/H235N |
|  | WLEK0336 | V15Q/S38T/L74F/M100K/C112S/S127T |
|  | WLEK0341 | V15Q/S38T/L74F/M100K/C112S/S127T |
|  | WLEK0353 | V15Q/S38T/L74F/M100K/C112S/S127T |
|  | WLEK0356 | V15Q/S38T/C112S/R124G/I135V/Y175F |
|  | WLEK0362 | V15Q/S38T/L74F/M100K/C112S/S127T/N169D |
|  | WLEK0374 | V15Q/S38T/L74F/M100K/C112S/S127T |
|  | WLEK0407 | V15Q/S38T/L74F/C112S |
|  | WLEK0427 | V15Q/M96L/C112S/R124G/N169D |
|  | WLEK0439 | V15Q/M96L/C112S |
|  | WLEK0479 | V15Q/S38T/C112S |
| L12: DNA-shuffling of additional top variants from libraries L1 – L4, L7, L10 and L11 | WLEK0244 | V15Q/I70V/E99D/C112S/I125V/H235N |
|  | WLEK0297 | P5'L/V15Q/C112S/Y175F |
|  | WLEK0342 | R8H/V15Q/C112S |
|  | WLEK0380 | V15Q/C112S/I135V/Y175F |
|  | WLEK0403 | V15Q/M48K/M96L/C112S/R124G |
|  | WLEK0415 | V15Q/M96L/C112S/R124G/N169D |
|  | WLEK0484 | V15Q/S38T/L74F/M100K/C112S/S127T/P162S |
|  | WLEK0503 | P5'L/V15Q/S38T/L74F/M100K/C112S/S127T/V180A;  t16a [T7 promo.] |
|  | WLEK0538 | P5'L/V15Q/C112S/Q160L/L191I |
|  | WLEK0549 | P5'L/V15Q/C112S/I135S/L200M |
|  | WLEK0553 | V15Q/C112S; c125t [pelB, silent] |

**Table S4 – Mutagenic primers used to shuffle with EK_L_ variant fragments from library L12**

| **Library & Description** | **Mutation(s)** | **Gene** | **Primer Sequence (5’ – 3’)** |
| --- | --- | --- | --- |
| L13: DNA-shuffling of L12 variants spiked with fragments encoding mutations observed previously at low frequency, and six newly observed random mutations. | T6'I | pelB | acctgctgccgatcgctgctgctg |
|  | I111V | EK_L_ | acggattacattcaaccagtgtctctgccggaagaaaac |
|  | E99A | EK_L_ | attgccatgatgcacttagcgatgaaagtgaactatacg |
|  | c68t | rbs | attttgtttaattttaagaagga |
|  | I128V/A129T | EK_L_ | ccaggtcgcatctgttccgtgaccggctggggagcgctt |
|  | I128V | EK_L_ | ccaggtcgcatctgttccgtggccggctggggagcgctt |
|  | I135T | EK_L_ | ccggctggggagcgcttacctatcaaggaagtact |
|  | g21a/a23t | lac op | cgactcactatagggaatttgtgagcggataacaattcccc |
|  | a23t | lac op | cgactcactataggggatttgtgagcggataacaattcccc |
|  | a81c | rbs | ctttaagaaggagctatacatatgaaatacctgctgcc |
|  | t69a | rbs | gaaataattttgtttaacattaagaaggagatatac |
|  | t71c | rbs | gaaataattttgtttaacttcaagaaggagatatacatatg |
|  | I75V | EK_L_ | gagacgcgacttgtggaccaaatagtaattaat |
|  | G27S | EK_L_ | gatgatcaacaggtctgcagcgcctctttagtgtctcgt |
|  | a5g | T7 promo. | gcgaaattaatgcgactcactat |
|  | A141T | EK_L_ | ggaagtactaccgatgtgttacaggaggcg |
|  | A141T/D142F | EK_L_ | ggaagtactacctttgtgttacaggaggcggat |
|  | D142F | EK_L_ | ggaagtactgcgtttgtgttacaggaggcggat |
|  | P214L | EK_L_ | gggtatcagtgtgcgttgctgaaccgtccaggtgtttat |
|  | A129T | EK_L_ | ggtcgcatctgttccataaccggctggggagcgctt |
|  | a76g | Rbs | gtttaactttaagagggagatatacatatgaaatacc |
|  | Q24L/G27S | EK_L_ | tattttgatgatcaactggtctgcagcgcctcttta |
|  | Q24L | EK_L_ | tattttgatgatcaactggtctgcggcgcctcttta |
|  | g21a | lac op. | tcactatagggaaattgtgagcg |
|  | I135T/Q137R | EK_L_ | tggggagcgcttacctatcgtggaagtact |
|  | Q137R | EK_L_ | tggggagcgcttatctatcgtggaagtact |

Table S5 – 321 unique EK_L_ variants discovered and their corresponding protein mutations.

| **Variant ID** | **Protein mutations (pelB-EK_L_-His6x)** | **DNA Mutations** |
| --- | --- | --- |
| WLEK0294 | V15Q/S38T/L74F/R82P/M100K/C112S/S127T/P162S |  |
| WLEK0296 | V15Q/R82P/C112S/P162S |  |
| WLEK0297 | P5'L/V15Q/R82P/C112S/Y175F |  |
| WLEK0298 | V15Q/R82P/N91S/C112S/I135V/Y175F | g127t [PelB,silent] |
| WLEK0303 | V15Q/R82P/M96L/C112S |  |
| WLEK0305 | S5F/V15Q/V56I/R82P/C112S |  |
| WLEK0306 | V15Q/S38T/L74F/R82P/C112S/A141V/P162S |  |
| WLEK0309 | V15Q/R82P/C112S/Y175F | c166t [EK] |
| WLEK0316 | Q17'H/V15Q/R82P/C112S |  |
| WLEK0321 | V15Q/R82P/C112S/I135V/Y175F |  |
| WLEK0322 | A11T/V15Q/R82P/C112S/I135V/Y175F |  |
| WLEK0323 | A11T/V15Q/R82P/C112S/I135V/Y175F |  |
| WLEK0324 | V15Q/S38T/L74F/R82P/M100K/C112S/S127T |  |
| WLEK0327 | V15Q/I75V/R82P/M96L/C112S/N169D | a377g,a440c,a659g |
| WLEK0328 | V15Q/R82P/C112S | g26a,t41c [lac op]; a79g [rbs] |
| WLEK0330 | V15Q/S38T/L74F/R82P/M100K/C112S/E115D/A141V/P162S |  |
| WLEK0333 | V15Q/M48K/R82P/C112S/Y175F |  |
| WLEK0334 | V15Q/R82P/C112S/I135V/Y175F |  |
| WLEK0335 | V15Q/I70V/R82P/E99D/C112S/I125V/H235N |  |
| WLEK0342 | R8H/V15Q/R82P/C112S |  |
| WLEK0343 | V15Q/R82P/C112S/E115D | g274a [EK] |
| WLEK0346 | V15Q/R82P/C112S/Y175F |  |
| WLEK0347 | V15Q/I70V/R82P/E99D/C112S/I125V/H235N |  |
| WLEK0348 | V15Q/R82P/C112S | t41a [lac op] |
| WLEK0349 | V15Q/R82P/C112S/V149I | c871t |
| WLEK0350 | L4'R/V15Q/R82P/C112S | t41a [lac op] |
| WLEK0358 | V15Q/R82P/C112S/I135V | c238t [EK] |
| WLEK0362 | V15Q/S38T/L74F/R82P/M100K/C112S/S127T/N169D |  |
| WLEK0369 | V15Q/R82P/C112S/R124G/I135V/Y175F |  |
| WLEK0389 | V15Q/M48K/R82P/C112S |  |
| WLEK0391 | V15Q/P50S/R82P/C112S |  |
| WLEK0393 | V15Q/R82P/C112S/R124G |  |
| WLEK0394 | V15Q/R82P/C112S/P224L |  |
| WLEK0395 | V15Q/R82P/C112S/N169D/M170del | a659g,t663g, g664del |
| WLEK0396 | V15Q/S38T/R82P/M100K/C112S |  |
| WLEK0397 | V15Q/S38T/R82P/C112S/S127T |  |
| WLEK0399 | V15Q/R82P/C112S/S127T/K156R | g18a |
| WLEK0403 | V15Q/M48K/R82P/M96L/C112S/R124G |  |
| WLEK0404 | V15Q/M48K/R82P/M96L/C112S |  |
| WLEK0405 | V15Q/R82P/C112S/N169D |  |
| WLEK0406 | L13'F/V15Q/G27D/R82P/C112S/R124G/N169D |  |
| WLEK0408 | V15Q/S38T/L74F/R82P/M100K/C112S/K156R |  |
| WLEK0409 | V15Q/S38T/L74F/R82P/M100K/C112S |  |
| WLEK0410 | V15Q/R82P/C112S/S127T | g316a [EK] |
| WLEK0411 | V15Q/L74F/R82P/C112S/S127T/K156R |  |
| WLEK0414 | V15Q/R82P/K86R/C112S | t528g [EK] |
| WLEK0415 | V15Q/R82P/M96L/C112S/R124G/N169D |  |
| WLEK0418 | V15Q/V43M/R82P/C112S |  |
| WLEK0419 | V15Q/S38T/L74F/R82P/C112S/K156R |  |
| WLEK0420 | V15Q/S38T/R82P/C112S/K156R |  |
| WLEK0421 | V15Q/L74F/R82P/M100K/C112S/S127T/K156R |  |
| WLEK0422 | V15Q/R82P/M100K/C112S/K156R | g613a [EK] |
| WLEK0423 | V15Q/T66M/R82P/C112S/K156R |  |
| WLEK0424 | V15Q/T66M/R82P/K156R |  |
| WLEK0430 | V15Q/R82P/C112S |  |
| WLEK0433 | V15Q/R82P/E99A/M100K/C112S/K156R |  |
| WLEK0435 | V15Q/S38T/L74F/R82P/M100K/C112S/S127T |  |
| WLEK0440 | V15Q/M48K/R82P/C112S/R124G/N169D |  |
| WLEK0443 | V15Q/S38T/R82P/C112S |  |
| WLEK0444 | T6'I/V15Q/S38T/R82P/M100K/C112S |  |
| WLEK0445 | V15Q/L74F/R82P/M100K/C112S |  |
| WLEK0446 | V15Q/R82P/C112S/S127T |  |
| WLEK0447 | V15Q/S38T/L74F/R82P/M100K/C112S/S127T | c409t [EK] |
| WLEK0448 | V15Q/S38T/L74F/R82P/M100K/C112S/S127T | c409t [EK] |
| WLEK0450 | V15Q/K54R/R82P/K86R/C112S |  |
| WLEK0452 | V15Q/R82P/C112S/R124G/N169D |  |
| WLEK0453 | V15Q/M48K/R82P/C112S/R124G/N169D/H242'Y |  |
| WLEK0454 | V15Q/R33C/R82P/C112S/R124G |  |
| WLEK0456 | V15Q/S38T/L74F/R82P/C112S | g610a [EK] |
| WLEK0457 | V15Q/L74F/R82P/M100K/C112S/S127T/K156R |  |
| WLEK0458 | V15Q/R82P/C112S/K156R |  |
| WLEK0460 | V15Q/R82P/C112S/P150S | t41a [lac op] |
| WLEK0463 | V15Q/R82P/M96L/C112S/N169D |  |
| WLEK0466 | V15Q/G27D/R82P/C112S/N169D/H245'N | g358a [EK] |
| WLEK0467 | V15Q/S38T/L74F/R82P/C112S/K156R/H246'* |  |
| WLEK0468 | V15Q/S38T/R82P/M100K/C112S/K156R |  |
| WLEK0470 | V15Q/R82P/C112S/H241'Y |  |
| WLEK0472 | V15Q/R82P/C112S/P150T |  |
| WLEK0473 | V15Q/M48K/R82P/C112S | g181a [EK] |
| WLEK0474 | V15Q/K54R/R82P/K86R/R88C/C112S |  |
| WLEK0477 | V15Q/S32T/R82P/C112S |  |
| WLEK0482 | V15Q/D76N/R82P/M100K/C112S/K156R |  |
| WLEK0485 | V15Q/S38T/L74F/R82P/M100T/C112S/I125V/H235N |  |
| WLEK0486 | V15Q/S38T/L74F/R82P/M100K/C112S/S127T/P162S/H235N | g367a [EK] |
| WLEK0487 | V15Q/S38T/R82P/C112S/R124G/I135V/Y175F |  |
| WLEK0488 | V15Q/S38T/L74F/R82P/M100K/C112S/S127T/P162S/H235N |  |
| WLEK0490 | V15Q/S38T/L74F/R82P/M100K/C112S/S127T | c289t [EK]; |
| WLEK0491 | V15Q/S38T/L74F/R82P/C112S/S127T |  |
| WLEK0492 | V15Q/S38T/L74F/R82P/M100K/C112S/S127T |  |
| WLEK0494 | V15Q/S38T/L74F/R82P/M100K/C112S/S127T/V204M/H235N |  |
| WLEK0495 | V15Q/S38T/I70V/R82P/E99D/C112S/S127T/N169D |  |
| WLEK0496 | V15Q/S38T/L74F/R82P/C112S/S127T |  |
| WLEK0498 | P5'L/V15Q/S38T/L74F/R82P/M100K/C112S/S127T/V180A | t16a [T7 promo]; |
| WLEK0499 | V15Q/S38T/L74F/R82P/E99D/C112S/I125V/N169D |  |
| WLEK0500 | V15Q/S38T/L74F/R82P/M100K/C112S/S127T/Y175F |  |
| WLEK0501 | V15Q/S38T/R82P/M100T/C112S/I125V/H235N |  |
| WLEK0502 | V15Q/S38T/L74F/R82P/M100K/C112S/S127T/N169D |  |
| WLEK0505 | V15Q/S38T/L74F/R82P/M100K/C112S/S127T/V204M/H235N |  |
| WLEK0507 | V15Q/S38T/L74F/R82P/M100K/C112S/S127T/N169D/L191I/L200M |  |
| WLEK0508 | V15Q/S38T/L74F/R82P/M100K/C112S/S127T/N169D/L191I/L200M | t718c,t724g,t733c,a736g,t755c,a757g,t763c [EK] |
| WLEK0509 | V15Q/S38T/I70V/R82P/C112S |  |
| WLEK0511 | V15Q/S38T/L74F/R82P/C112S |  |
| WLEK0512 | V15Q/S38T/R82P/M100K/C112S/S127T |  |
| WLEK0513 | V15Q/S38T/L74F/R82P/M100K/C112S/S127T/N169D/L200M |  |
| WLEK0514 | V15Q/S38T/R82P/M100K/C112S/S127T |  |
| WLEK0515 | V15Q/S38T/R82P/E99D/C112S/I125V/P214S/H235N |  |
| WLEK0516 | V15Q/I70V/R82P/M100T/C112S/I125V/H246'* | c890t,a891g,c892a [His]; |
| WLEK0517 | V15Q/S38T/L74F/R82P/M100K/C112S/S127T/N169D |  |
| WLEK0518 | V15Q/S38T/L74F/R82P/M100K/C112S/Q160L |  |
| WLEK0520 | V15Q/R82P/M96L/C112S/S127T |  |
| WLEK0521 | V15Q/S38T/L74F/R82P/M100K/C112S/S127T/I135S/G179S |  |
| WLEK0522 | V15Q/S38T/L74F/R82P/M100K/C112S/A141V/P162S |  |
| WLEK0523 | P5'L/V15Q/R82P/M100K/C112S/S127T/N169D/L191I |  |
| WLEK0524 | V15Q/S38T/R82P/C112S | c670t [EK]; |
| WLEK0526 | V15Q/S38T/L74F/R82P/M100K/C112S/S127T/Q160L |  |
| WLEK0528 | V15Q/S38T/L74F/R82P/M100K/C112S/Q160L/V180A/L191I/L200M |  |
| WLEK0529 | V15Q/R82P/M96L/C112S/R124G |  |
| WLEK0530 | V15Q/S38T/L74F/R82P/M100K/C112S/A141V/P162S |  |
| WLEK0536 | V15Q/I70V/R82P/M100K/C112S/S127T |  |
| WLEK0538 | P5'L/V15Q/R82P/C112S/Q160L/L191I |  |
| WLEK0539 | V15Q/R82P/M100K/C112S/S127T |  |
| WLEK0540 | V15Q/R82P/C112S/Q160L/N169D/H246'* | c890t,a891g,c892a [His]; |
| WLEK0541 | V15Q/I70V/R82P/M100T/C112S/I125V/N169D | g21a [lac op]; |
| WLEK0542 | V15Q/L74F/R82P/M100K/C112S/S127T |  |
| WLEK0546 | V15Q/I75V/R82P/M96L/C112S/N169D |  |
| WLEK0547 | V15Q/R82P/C112S/Q160L/V180A/H246'* | c890t,a891g,c892a [His]; |
| WLEK0550 | P5'L/V15Q/R82P/C112S/Q160L/N165D/H246'* | c68t [rbs]; c890t,a891g,c892a [His]; |
| WLEK0551 | P5'L/V15Q/R82P/C112S/Q160L |  |
| WLEK0552 | V15Q/R82P/C112S | t42c [lac op]; c125t [PelB]; |
| WLEK0553 | V15Q/R82P/C112S | c125t [PelB]; |
| WLEK0554 | P5'L/V15Q/R82P/C112S/L200M | t25a [lac op]; |
| WLEK0555 | P5'L/V15Q/R82P/C112S |  |
| WLEK0556 | V15Q/R82P/E99D/C112S/S127T/N169D | g583a [EK]; |
| WLEK0557 | M20'I/V15Q/R82P/C112S | c125t [PelB]; |
| WLEK0558 | P5'L/V15Q/D22E/R82P/C112S/Q160L | t16a [T7 promo]; t25a [lac op]; |
| WLEK0559 | V15Q/R82P/C112S/Q160L |  |
| WLEK0563 | R8H/V15Q/L74F/R82P/M100K/C112S | c238t [EK]; |
| WLEK0564 | V15Q/R82P/C112S | c323t [EK]; |
| WLEK0566 | V15Q/R82P/C112S/V180A |  |
| WLEK0570 | V15Q/R82P/C112S/Q160L/N165D |  |
| WLEK0571 | V15Q/R82P/C112S |  |
| WLEK0573 | P5'L/V15Q/R82P/C112S | t16a [T7 promo]; t25a [lac op]; |
| WLEK0575 | V15Q/R82P/C112S | t42c [lac op]; c125t [PelB]; |
| WLEK0576 | P5'L/V15Q/D21N/R82P/C112S/L200M |  |
| WLEK0580 | P5'L/V15Q/D21N/R82P/C112S/V180A/L200M |  |
| WLEK0581 | V15Q/R82P/C112S/V180A/L200M |  |
| WLEK0583 | V15Q/R82P/M96L/C112S/R124G/N169D/R222Q |  |
| WLEK0585 | V15Q/R82P/C112S/L200M |  |
| WLEK0586 | V15Q/R82P/C112S | t25a [lac op]; |
| WLEK0588 | V15Q/R82P/C112S/L200M | c130t [PelB]; |
| WLEK0592 | V15Q/R82P/C112S | t42c [lac op]; c125t [PelB]; |
| WLEK0598 | V15Q/D21N/R82P/C112S/L191I/L200M |  |
| WLEK0601 | V15Q/S63N/R82P/C112S |  |
| WLEK0603 | V15Q/R82P/C112S | t42c [lac op]; |
| WLEK0604 | V15Q/R82P/C112S | a12g [T7 promo]; t42c [lac op]; c125t [PelB]; |
| WLEK0606 | P5'L/R8H/V15Q/R82P/C112S | t16a [T7 promo]; |
| WLEK0610 | M20'I/V15Q/R82P/C112S | a12g [T7 promo]; t42c [lac op]; c125t [PelB]; |
| WLEK0611 | V15Q/L74F/R82P/C112S | c374t [EK]; |
| WLEK0615 | P5'L/V15Q/D21N/D22E/R82P/C112S |  |
| WLEK0621 | V15Q/R82P/C112S/Q160L/P162S | c436t,t625c [EK] |
| WLEK0622 | V15Q/R82P/Y84H/C112S/Q160L |  |
| WLEK0623 | V15Q/R82P/C112S | a12g [T7 promo]; t42c [lac op]; |
| WLEK0624 | P5'L/V15Q/D22E/R82P/C112S |  |
| WLEK0625 | V15Q/D22E/R82P/C112S |  |
| WLEK0627 | V15Q/R82P/R88H/C112S |  |
| WLEK0628 | P5'L/V15Q/R82P/C112S/V180A/L191I |  |
| WLEK0629 | V15Q/D21N/D22E/R82P/C112S/L200M |  |
| WLEK0630 | P5'L/V15Q/D22E/S63N/R82P/C112S | t16a [T7 promo]; t25a [lac op]; a223g,c229g,c334t,c340g,t346c,g352c,a550c [EK] |
| WLEK0631 | V15Q/R82P/C112S | c229t [EK] |
| WLEK0632 | P5'L/V15Q/R82P/C112S | g80t [rbs]; |
| WLEK0633 | M20'I/V15Q/R82P/C112S | a12g [T7 promo]; t42c [lac op]; |
| WLEK0637 | P5'L/V15Q/R82P/Y84H/C112S | t16a [T7 promo]; t25a [lac op]; |
| WLEK0638 | V15Q/R82P/C112S/T167M | c654t [EK]; |
| WLEK0639 | M20'I/V15Q/R82P/C112S | a12g [T7 promo]; |
| WLEK0641 | V15Q/R82P/C112S/Q158H/Q160L | c640g [EK] |
| WLEK0642 | P5'L/V15Q/R82P/Y84H/C112S | g352c,t397c,c403t,c415t [EK] |
| WLEK0644 | P5'L/V15Q/R82P/Y84H/C112S/L200M |  |
| WLEK0650 | P5'L/V15Q/V43M/R82P/C112S | t25a [lac op]; |
| WLEK0652 | V15Q/S29F/S38T/L74F/R82P/M100K/C112S/S127T |  |
| WLEK0653 | V15Q/R82P/M96L/C112S/Q158* |  |
| WLEK0654 | M20'I/V15Q/R82P/C112S/G130D | t42c [lac op]; |
| WLEK0663 | V15Q/R82P/M96L/C112S/R124G/G188D |  |
| WLEK0666 | M20'I/V15Q/R82P/C112S/S182N | a12g [T7 promo]; t42c [lac op]; |
| WLEK0673 | V15Q/R82P/C112S/L239'H/E240'L/H241'L/H242'F/H243'I/H244'K/H245'I/H246'Q |  |
| WLEK0674 | P5'L/V15Q/S38T/L74F/R82P/M100K/C112S/S127T | g226a [EK]; |
| WLEK0675 | P5'L/V15Q/S38T/R82P/C112S | g226a [EK]; |
| WLEK0676 | P5'L/V15Q/S38T/L74F/R82P/M100K/C112S/S127T/V180A | g226a,c685g,t688c,c697t [EK]; |
| WLEK0679 | P5'L/V15Q/S38T/L74F/R82P/M100K/C112S/S127T/V180A | c685g,t688c,c697t [EK]; |
| WLEK0683 | P5'L/V15Q/S38T/L74F/R82P/M100K/C112S/S127T/V180A | g226a,c511t,c685g,t688c,c697t [EK]; |
| WLEK0684 | V15Q/S38T/L74F/R82P/M100K/C112S/S127T/Y175F/H235N |  |
| WLEK0685 | P5'L/V15Q/S38T/L74F/R82P/M100K/C112S/I135V/Y175F | g226a [EK]; |
| WLEK0686 | V15Q/M48K/R82P/M96L/C112S/I135S/L200M | t556g,c640g,t755c,a757g [EK]; |
| WLEK0691 | A16'P/V15Q/S38T/I75V/R82P/E99A/I111V/C112S/A129T/I135T | t69a [rbs]; |
| WLEK0692 | V15Q/S38T/M48K/R82P/C112S/I135S/L200M | t556g,c640g,t755c,a757g [EK]; |
| WLEK0693 | P5'L/A19'S/V15Q/S38T/L74F/R82P/M100K/C112S/S127T/Y175F | g226a [EK] |
| WLEK0694 | V15Q/S38T/R82P/C112S/Q160L | a631g,c640g,g706a [EK]; |
| WLEK0695 | V15Q/S38T/L74F/R82P/M100K/C112S/S127T | g226a [EK]; |
| WLEK0696 | P5'L/V15Q/M48K/R82P/M96L/C112S/I135S | g148t [PelB]; t556g [EK]; |
| WLEK0697 | V15Q/S38T/L74F/R82P/M100K/C112S/I135S/Y175F | t556g,c640g [EK]; |
| WLEK0699 | P5'L/A19'V/V15Q/R82P/E99A/C112S/A129T/I135S | a23t [lac op]; t71c [rbs]; c125t [PelB]; t556g [EK]; |
| WLEK0700 | V15Q/I75V/R82P/M96L/E99A/C112S/I128V/A129T/I135T |  |
| WLEK0702 | V15Q/S38T/L74F/R82P/M100K/C112S/S127T/P162S | t16a [T7 promo]; |
| WLEK0703 | V15Q/S38T/L74F/R82P/C112S/I135V | g226a [EK]; |
| WLEK0705 | V15Q/S38T/L74F/R82P/M100K/C112S/S127T/L200M | t453a,g454a,c640g,t755c,a757g [EK]; |
| WLEK0706 | P5'L/V15Q/S38T/L74F/R82P/C112S/Q160L | t16a [T7 promo]; a631g,c640g [EK]; |
| WLEK0707 | V15Q/I70V/R82P/M96L/C112S/I135S | t556g [EK]; |
| WLEK0708 | P5'L/A16'P/V15Q/S38T/I75V/R82P/E99A/C112S/I128V/I135T/Q137R/Q160L | a23t [lac op]; t71c [rbs]; a631g,c640g [EK]; |
| WLEK0709 | P5'L/V15Q/R82P/C112S/I135S | t205g,c208g,t556g,c640g [EK]; |
| WLEK0710 | V15Q/S38T/R82P/C112S/Y175F |  |
| WLEK0711 | P5'L/V15Q/R33H/R82P/C112S/I135S/Q160L/Y175F | t205g,c208g,t556g,a631g,c640g [EK]; |
| WLEK0713 | V15Q/Q24L/R82P/E99A/C112S/I128V/A129T/I135T/Q137R/H235N | t71c [rbs]; |
| WLEK0715 | V15Q/Q24L/G27S/S38T/R82P/E99A/C112S/S127T/Q137R/L200M | t755c,a757g [EK]; |
| WLEK0716 | V15Q/S38T/L74F/R82P/C112S/Y175F | c374t [EK]; |
| WLEK0717 | T6'I/V15Q/Q24L/R82P/C112S/A129T/I135T | messy[T7 promo]; g21a,a23t [lac op]; a76g [rbs]; c125t [PelB]; |
| WLEK0719 | V15Q/S38T/L74F/R82P/M100K/C112S/S127T/P162S | c235t [EK]; |
| WLEK0721 | P5'L/A16'P/V15Q/R82P/E99A/C112S/A129T/Q160L | a23t [lac op]; t69a,a81c [rbs]; a631g [EK]; |
| WLEK0722 | P5'L/V15Q/S38T/L74F/R82P/C112S/Y175F | t16a [T7 promo]; g226a [EK]; |
| WLEK0727 | P5'L/V15Q/R82P/M100K/C112S/S127T | t205g,c208g,g370t [EK]; |
| WLEK0728 | V15Q/M48K/R82P/M96L/C112S/Q160L/Y175F | a631g,c640g,g760a [EK]; |
| WLEK0729 | V15Q/S38T/R82P/C112S/Y175F | g226a [EK]; |
| WLEK0732 | P5'L/V15Q/R82P/C112S/I135S | g74a [rbs]; t205g,c208g,t556g,c640g [EK]; |
| WLEK0733 | P5'L/V15Q/I70V/R82P/C112S/I135S/N169D | t556g [EK]; |
| WLEK0734 | V15Q/S38T/I75V/R82P/E99A/C112S/I128V/I135T/Y175F |  |
| WLEK0735 | P5'L/V15Q/R82P/C112S/I135S/L200M | c104t, t205g,c208g,t556g,c640g,t755c,a757g [EK]; |
| WLEK0736 | V15Q/S38T/L74F/R82P/M100K/C112S/S127T/V180A | g226a,c685g,t688c,c697t [EK]; |
| WLEK0737 | P5'L/V15Q/S38T/T66M/L74F/R82P/M100K/C112S/S127T/P162S | t205g,c208g,g226a [EK]; |
| WLEK0738 | P5'L/V15Q/R82P/C112S/I135S | t556g,c640g [EK]; |
| WLEK0739 | V15Q/L74F/R82P/M100K/C112S/I135V/H235N |  |
| WLEK0740 | P5'L/A16'V/V15Q/S38T/L74F/R82P/M100K/C112S/S127T/V180A | g226a,c685g,t688c,c697t [EK]; |
| WLEK0741 | P5'L/V15Q/S38T/L74F/R82P/M100K/C112S/S127T/V180A | t16a [T7 promo]; g226a,c685g,t688c,c697t [EK]; |
| WLEK0743 | P5'L/V15Q/R82P/M96L/C112S/I135V/Y175F | t205g,c208g [EK]; |
| WLEK0745 | P5'L/V15Q/R82P/C112S/I135S/Q160L/L200M | t556g,a631g,c640g,t755c,a757g [EK]; |
| WLEK0746 | P5'L/V15Q/R82P/R88S/C112S/I135S | g199a,t556g,c640g [EK]; |
| WLEK0747 | P5'L/V15Q/R82P/C112S/I135S/L200M | t205g,c208g,t556g,c640g,t755c,a757g [EK]; |
| WLEK0748 | V15Q/S38T/L74F/R82P/M100K/C112S/S127T/V180A | g226a,g613a,c685g,t688c,c697t [EK]; |
| WLEK0749 | P5'L/V15Q/I70V/R82P/M100K/C112S/S127T/P162S |  |
| WLEK0750 | V15Q/S38T/R82P/M96L/C112S/S127T/P162S |  |
| WLEK0752 | A16'P/V15Q/G27S/S38T/R82P/E99A/I111V/C112S/I128V/A129T | t69a [rbs]; c125t [PelB]; |
| WLEK0753 | V15Q/S38T/L74F/R82P/M96L/C112S/R124G/N169D |  |
| WLEK0754 | P5'L/V15Q/R82P/C112S/I135S | t205g,c208g,t556g,c640g [EK]; |
| WLEK0755 | V15Q/Q24L/G27S/I75V/R82P/E99A/C112S/I135T | a23t [lac op]; t71c [rbs]; |
| WLEK0756 | V15Q/S38T/L74F/R82P/C112S/Q160L/L191I | c374t,a631g,c640g,t709c,t718c,t724g,t733c,a736g [EK]; |
| WLEK0758 | P5'L/V15Q/S38T/L74F/R82P/M100K/C112S/R124G | g226a,c640g [EK]; |
| WLEK0759 | V15Q/R82P/C112S/I135S/N169D | t205g,c208g,t556g [EK]; |
| WLEK0760 | V15Q/S38T/L74F/R82P/C112S/I135V/P162S | g226a [EK]; |
| WLEK0761 | P5'L/V15Q/R82P/C112S/I135S | t205g,c208g,c535t,c554t,t556g,c640g [EK]; |
| WLEK0762 | P5'L/V15Q/R82P/C112S/I135S | t16a [T7 promo]; t205g,c208g,t556g [EK]; |
| WLEK0763 | V15Q/I70V/R82P/C112S/I135S | t556g,c640g [EK]; |
| WLEK0764 | R8H/V15Q/Q24L/R82P/E99A/C112S/I128V/A129T/I135T | t71c [rbs]; |
| WLEK0765 | P5'L/V15Q/I70V/R82P/E99D/C112S/I125V/H235N | a451t [EK]; |
| WLEK0766 | P5'L/V15Q/M48K/R82P/M96L/C112S/R124G |  |
| WLEK0767 | P5'L/V15Q/S38T/L74F/R82P/R88C/M100K/C112S/S127T/N169D | t16a [T7 promo]; g226a [EK]; |
| WLEK0768 | P5'L/V15Q/I70V/R82P/E99D/C112S/I135V/Y175F | a451t [EK]; |
| WLEK0771 | P5'L/A7'T/V15Q/M48K/R82P/M96L/C112S/R124G |  |
| WLEK0772 | P5'L/V15Q/S38T/R82P/C112S/R124G/V180A/H235N | t16a [T7 promo]; g226a,c685g,t688c,c697t [EK]; |
| WLEK0774 | P5'L/L14'F/V15Q/R82P/M96L/C112S/R124G/Y175F | t205g,c208g [EK]; |
| WLEK0775 | V15Q/L74F/R82P/M100K/C112S/S127T/P162S/H235N |  |
| WLEK0776 | V15Q/R82P/C112S/Q160L | a631g,c640g [EK]; |
| WLEK0778 | V15Q/Q24L/G27S/R82P/E99A/C112S/I128V/A129T/Q137R | a23t [lac op]; t71c [rbs]; |
| WLEK0779 | V15Q/R82P/M96L/C112S/R124G/N169D |  |
| WLEK0780 | V15Q/R82P/E99A/C112S/I128V/I135T/Q137R/N169D/L191I/H235N | t69a [rbs]; t709c,t718c,t724g,t733c,a736g [EK]; |
| WLEK0781 | V15Q/G27S/I75V/R82P/E99A/C112S/I128V/A129T/I135T/V180A | c640g,c685g,t688c,c697t [EK]; |
| WLEK0783 | V15Q/R82P/M100K/C112S/S127T/P162S |  |
| WLEK0784 | P5'L/V15Q/Q24L/R82P/M96L/C112S/R124G/L200M | c640g,t755c,a757g [EK]; |
| WLEK0785 | P5'L/V15Q/S38T/R82P/M96L/C112S/R124G/I135V/Y175F/L200M | t16a [T7 promo]; t755c,a757g [EK]; |
| WLEK0786 | V15Q/S38T/L74F/R82P/M100K/C112S/S127T/P162S | g77a [rbs]; |
| WLEK0787 | T6'I/V15Q/Q24L/R82P/E99A/C112S/A129T/I135T/D142F | g21a,a23t [lac op]; |
| WLEK0789 | P5'L/V15Q/R82P/C112S/I135V/Y175F |  |
| WLEK0790 | V15Q/R82P/C112S/I135S/L200M | t556g,c640g,t755c,a757g [EK]; |
| WLEK0792 | P5'L/V15Q/R82P/C112S/I128V/A129T | g21a,a23t [lac op]; t71c [rbs]; |
| WLEK0793 | P5'L/V15Q/R82P/C112S/S127T/P162S | g30a [lac op]; t205g,c208g [EK]; |
| WLEK0794 | P5'L/V15Q/R82P/M96L/C112S/S127T/V180A/L200M | t205g,c208g,g655a,c685g,t688c,c697t,t755c,a757g [EK]; |
| WLEK0795 | V15Q/I70V/R82P/E99D/C112S/I125V | a451t [EK]; |
| WLEK0796 | V15Q/I70V/R82P/C112S/Y175F |  |
| WLEK0797 | P5'L/V15Q/R82P/C112S/R124G/N169D | t205g,c208g [EK]; |
| WLEK0799 | P5'L/V15Q/R82P/C112S/S127T/P162S |  |
| WLEK0802 | A16'P/V15Q/G27S/I70V/R82P/E99D/C112S/I125V/H235N | g21a,a23t [lac op]; g304a,a451t [EK] |
| WLEK0803 | P5'L/V15Q/R82P/C112S/Y175F | t205g,c208g,g790a [EK]; |
| WLEK0804 | P5'L/V15Q/R82P/C112S |  |
| WLEK0806 | P5'L/V15Q/R82P/C112S/Q160L/L191I | t16a [T7 promo]; a631g,c640g,t709c,t718c,t724g,t733c,a736g [EK]; |
| WLEK0807 | V15Q/R82P/C112S/I135V |  |
| WLEK0808 | P5'L/V15Q/I70V/R82P/E99D/C112S/I125V/Y175F | t205g,c208g,a451t, [EK]; |
| WLEK0810 | P5'L/V15Q/R82P/C112S/I135V/Y175F | t16a [T7 promo]; |
| WLEK0814 | P5'L/V15Q/R82P/M100K/C112S/R124G/L200M | g124a [PelB]; t755c,a757g [EK]; |
| WLEK0815 | P5'L/V15Q/R82P/E99D/C112S/I135V/Y175F | a451t [EK]; |
| WLEK0816 | P5'L/V15Q/R82P/C112S/Q160L/L191I | a631g,c640g,t709c,t718c,t724g,t733c,a736g [EK]; |
| WLEK0818 | V15Q/Q24L/L74F/R82P/I111V/C112S/I125V/I135T/Q137R/H235N | g21a,a23t [lac op]; t69a [rbs]; |
| WLEK0819 | V15Q/I70V/R82P/C112S/Q160L/L191I | a631g,c640g,t709c,t718c,t724g,t733c,a736g [EK]; |
| WLEK0820 | P5'L/V15Q/R82P/C112S/Y175F | g331a [EK]; |
| WLEK0821 | P5'L/V15Q/R82P/C112S/I128V/Q160L/L191I | t205g,c208g,a631g,c640g,t709c,t718c,t724g,t733c,a736g [EK]; |
| WLEK0826 | P5'L/V15Q/R82P/C112S/I135V/P162S |  |
| WLEK0827 | V15Q/R82P/M100K/C112S/V180A | t453a,g454a,c685g,t688c,c697t [EK]; |
| WLEK0828 | V15Q/Q24L/G27S/I75V/R82P/E99A/C112S/A129T/L191I | g21a,a23t [lac op]; t71c [rbs]; t724g [EK]; |
| WLEK0829 | V15Q/Q24L/I70V/I75V/R82P/C112S/I135S/D142F/L200M | a23t [lac op]; t69a [rbs]; t205g,c208g,t556g,t755c,a757g [EK]; |
| WLEK0830 | V15Q/I70V/R82P/E99D/C112S/R124G/N169D | t16a [T7 promo]; a451t [EK]; |
| WLEK0831 | V15Q/Q24L/I70V/I75V/R82P/I111V/C112S/I135S/Q137R/L200M | g21a,a23t [lac op]; t71c [rbs]; t556g,t755c,a757g [EK]; |
| WLEK0833 | V15Q/R82P/E99A/C112S/I128V/I135T/D142F/N169D/ | a23t [lac op]; t71c [rbs]; |
| WLEK0834 | P5'L/A16'P/V15Q/G27S/R82P/E99A/I111V/C112S/I128V/Y175F | t71c [rbs]; |
| WLEK0835 | P5'L/V15Q/R82P/C112S/I135S/A147V/L200M | t205g,c208g,t556g,c640g,t755c,a757g [EK]; |
| WLEK0836 | P5'L/V15Q/R82P/M96L/C112S/I135S/R216H | t556g,c640g [EK]; |
| WLEK0837 | V15Q/Q24L/G27S/S38T/L74F/R82P/E99A/C112S/I128V/I135T/D142F | g21a,a23t [lac op]; a76g [rbs]; c125t [PelB]; c640g [EK]; |
| WLEK0838 | P5'L/V15Q/G27S/I75V/R82P/M100K/I111V/C112S/I128V/A129T/Y175F/L200M | t69a,a81c [rbs]; t709c,t755c,a757g [EK]; |
| WLEK0839 | P5'L/V15Q/R82P/C112S/P162S |  |
| WLEK0840 | V15Q/D22N/Q24L/I75V/R82P/C112S/I128V/A129T/I135T/D142F/Y175F | t16a [T7 promo]; t71c [rbs]; t205g,c208g [EK]; |
| WLEK0841 | P5'L/V15Q/A40S/I70V/R82P/M96L/C112S/R124G/N169D |  |
| WLEK0842 | P5'L/V15Q/Q24L/G27S/R82P/E99A/C112S/I128V/A129T/D142F | t69a [rbs]; c125t [PelB]; |
| WLEK0843 | P5'L/V15Q/R82P/C112S/P162S | t16a [T7 promo]; |
| WLEK0845 | P5'L/V15Q/L74F/R82P/E99D/C112S/I135V/M170I/Y175F | a451t,g664t [EK]; |
| WLEK0846 | V15Q/M48K/L74F/R82P/E99A/I111V/C112S/D142F/P214L |  |
| WLEK0847 | V15Q/G27S/R82P/C112S | t16a [T7 promo]; a76g [rbs]; c125t [PelB]; |
| WLEK0848 | V15Q/Q24L/G27S/R82P/C112S/I128V/I135S/L200M/P214L | a23t [lac op]; t205g,c208g,t556g,t755c,a757g [EK]; |
| WLEK0849 | P5'L/V15Q/R82P/E99A/C112S/I128V/I135S/D142F/Q160L/P214L | g21a,a23t [lac op]; t71c [rbs]; t556g,a631g,c640g [EK]; |
| WLEK0850 | P5'L/V15Q/R82P/C112S/I125V/A173V/H235N |  |
| WLEK0851 | P5'L/V15Q/R82P/C112S/P114S/I135V/Y175F | c208t [EK]; |
| WLEK0852 | V15Q/Q24L/G27S/R82P/I111V/C112S/I125V/A129T/D142F | t69a [rbs]; |
| WLEK0853 | T6'I/V15Q/R82P/E99A/K101N/C112S/I128V/I135T/A141T/D142F/N169D | t71c [rbs]; |
| WLEK0854 | V15Q/Q24L/G27S/R82P/I111V/C112S/I125V/A129T/D142F/Q160L/Y175F/N169D | t69a [rbs]; |
| WLEK0855 | V15Q/I70V/R73STOP/R82P/E99D/C112S/I125V/H235N | a451t [EK]; |
| WLEK0856 | P5'L/V15Q/A28V/R82P/C112S/Y175F |  |
| WLEK0857 | V15Q/G27S/R82P/E99T/C112S/A129T/I135V/A141T/D142F | t71c [rbs]; |
| WLEK0858 | T6'I/A16'P/V15Q/Q24L/R82P/C112S/I125V/I135T/D142F/V180A/P214L | t69a,a81c [rbs]; c125t [PelB]; c685g,t688c,c697t [EK]; |

**A**


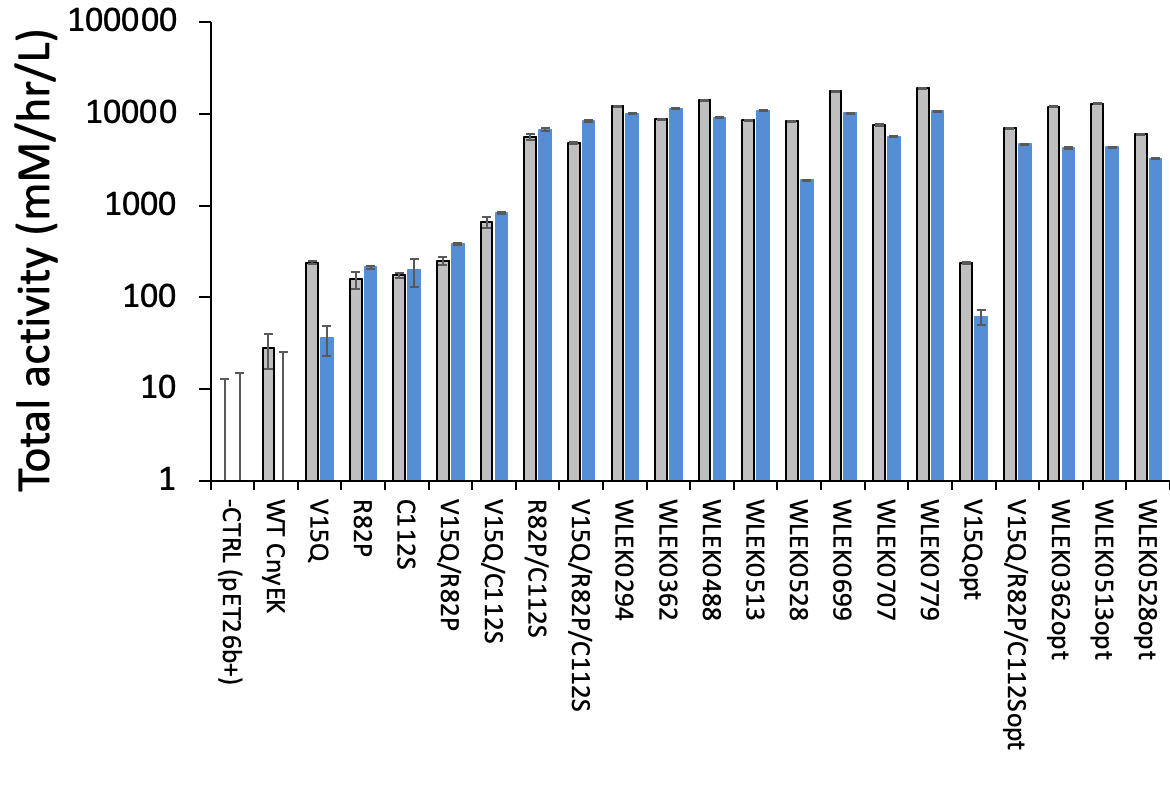


**B**


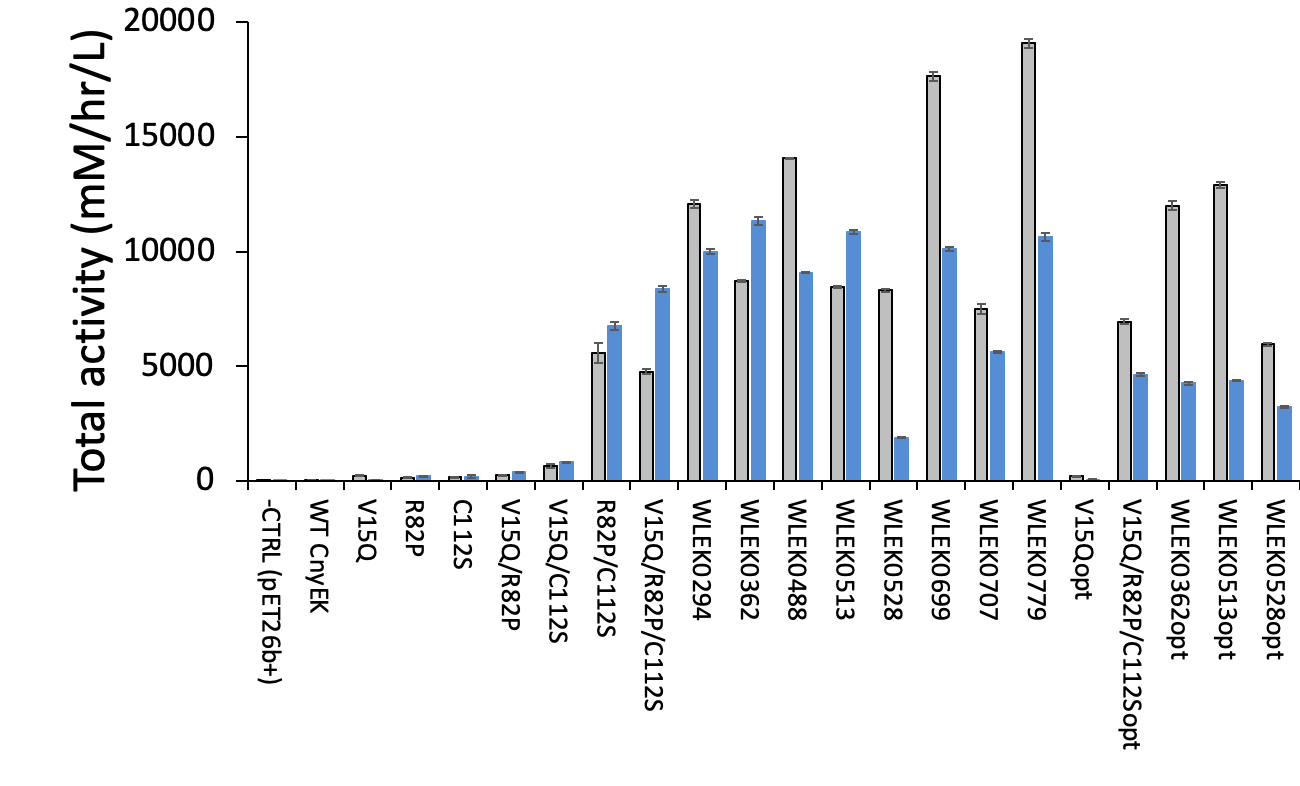


**Figure S4. Evolution of total activity in the variants retested after expression at 30** °**C (grey bars) and 37** °**C (blue bars).** The same data are plotted as A) log-scale activity and B) linear-scale activity, to allow comparisons across the full range

*Partial least squares regression.*

**A**


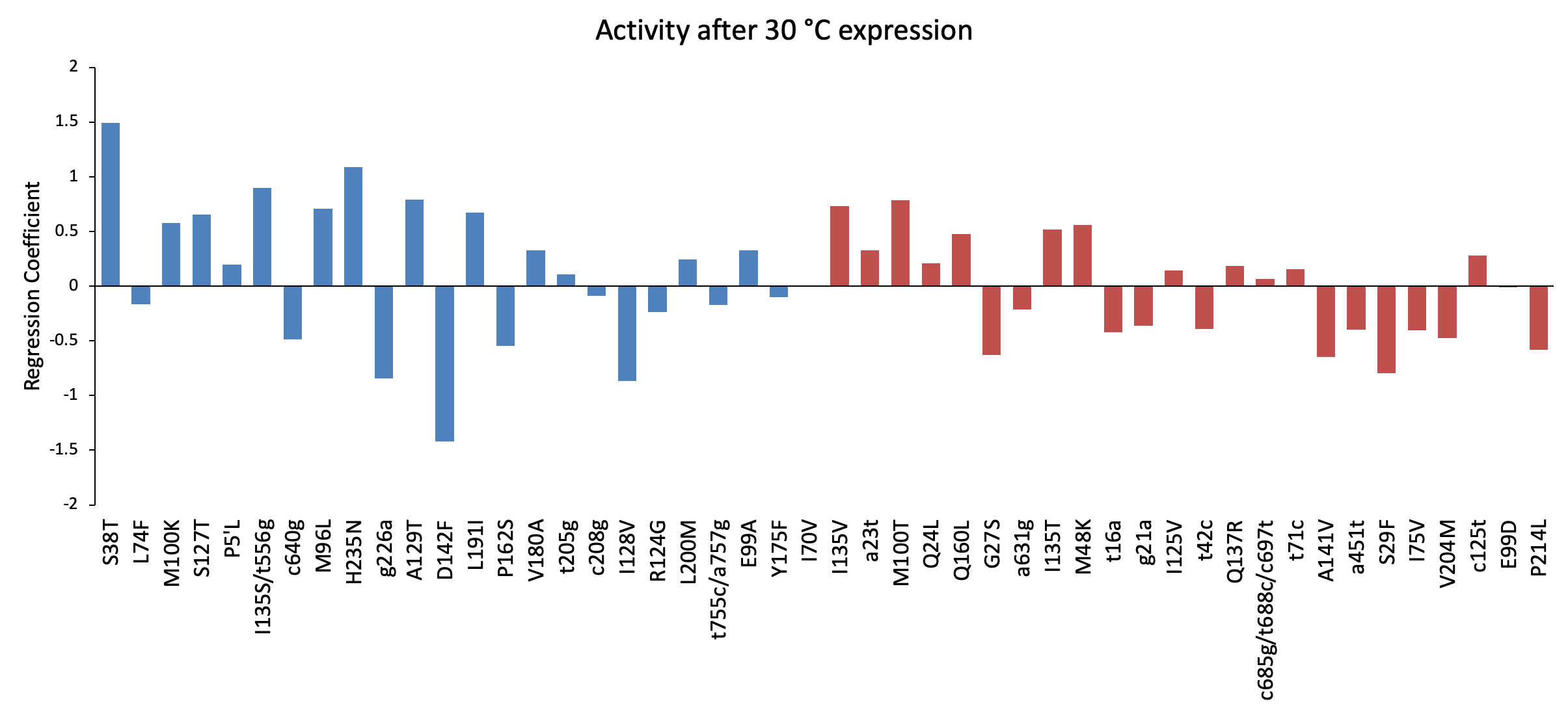


**B**


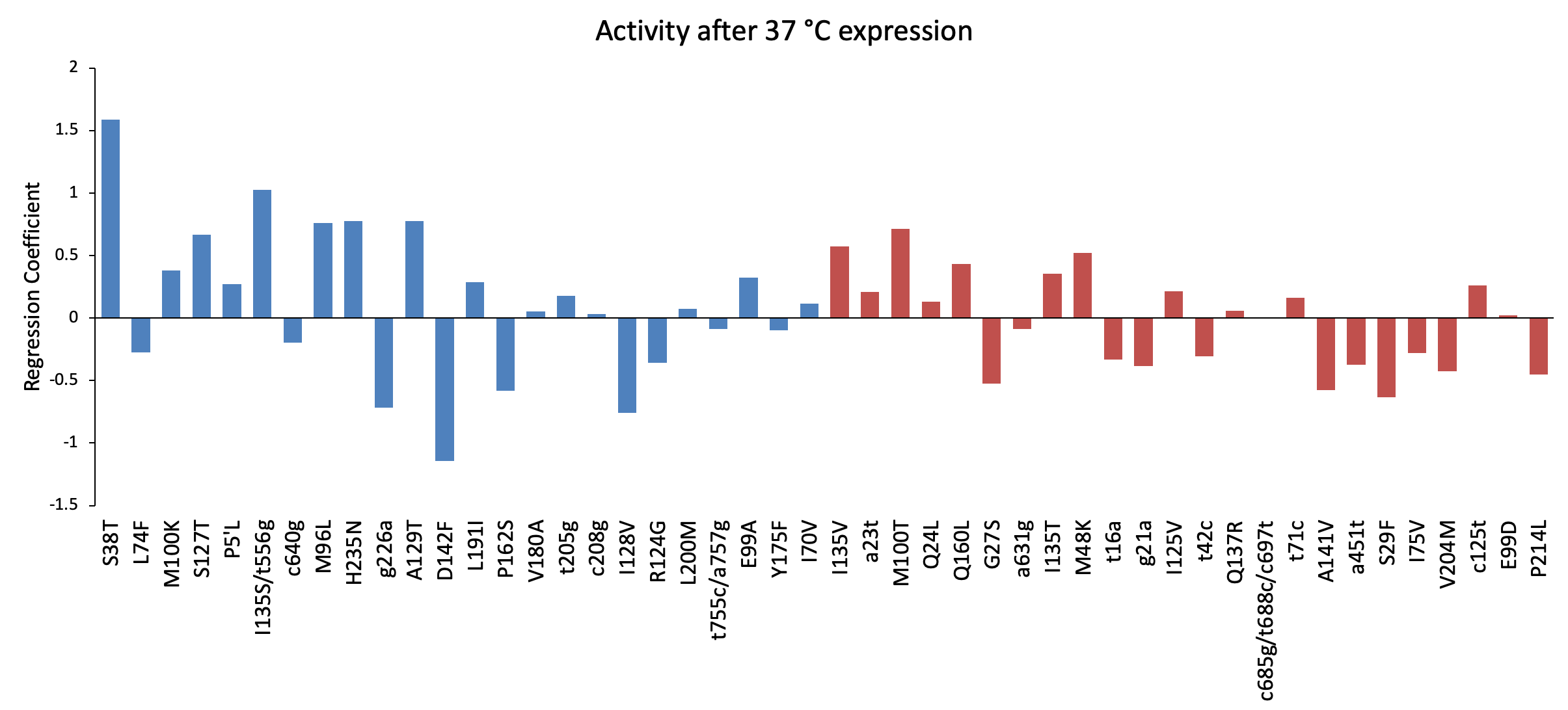


**C**


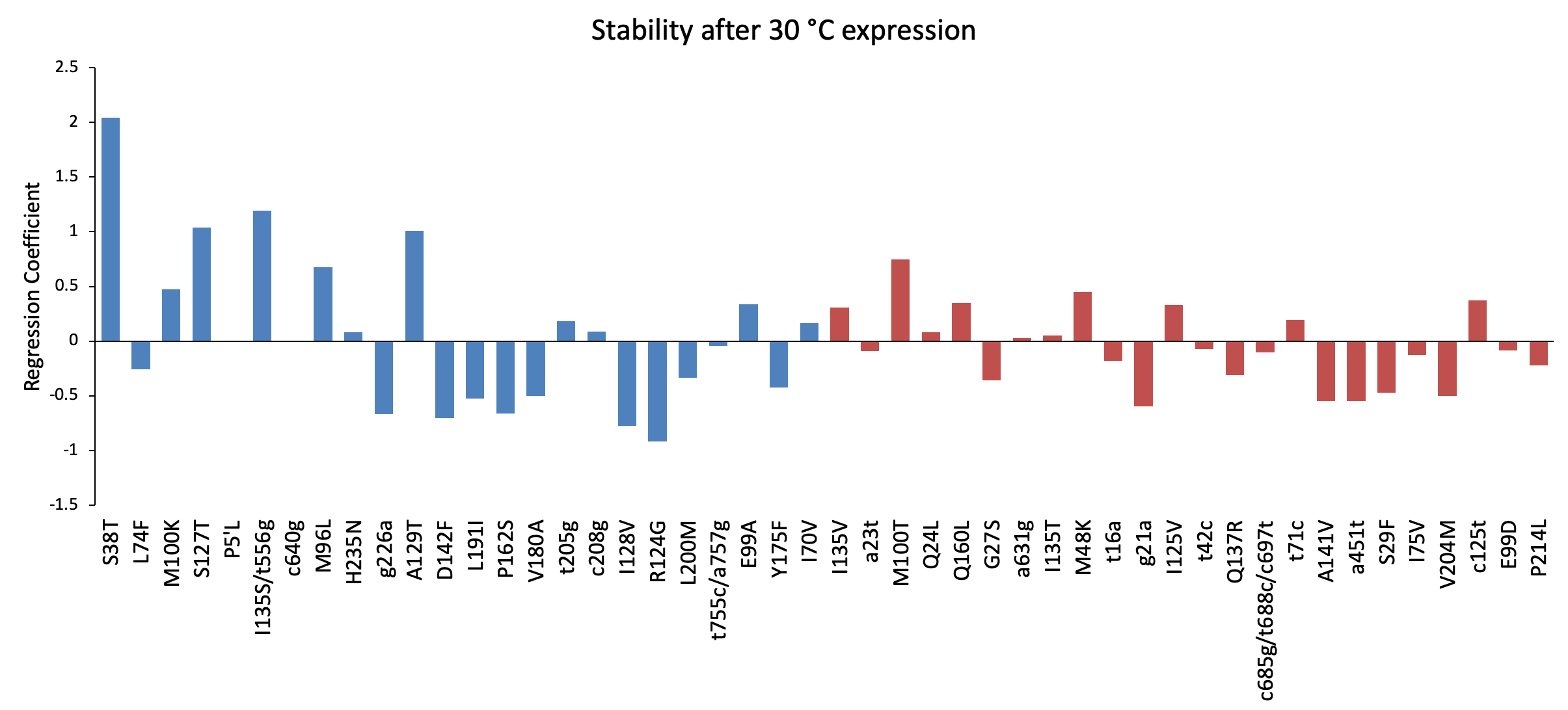


**D**


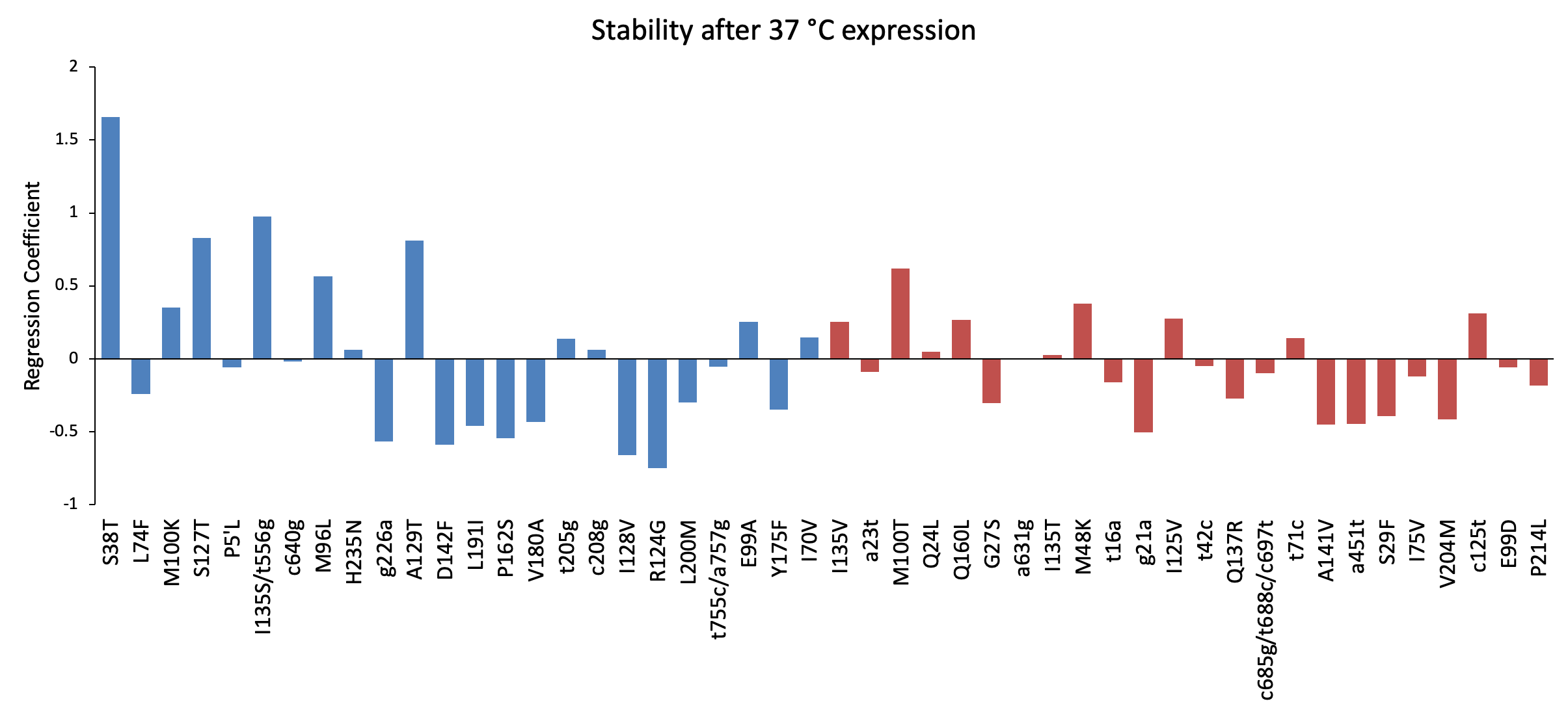


**E F**


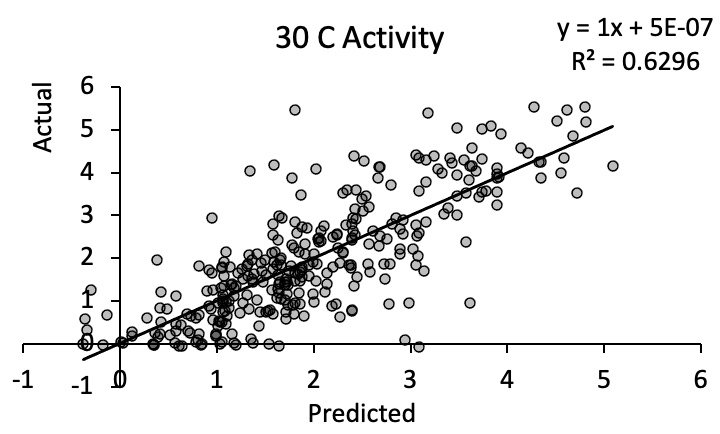

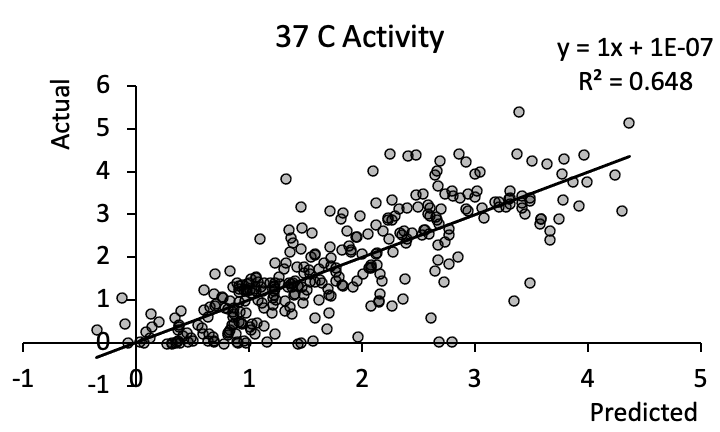


**G H**


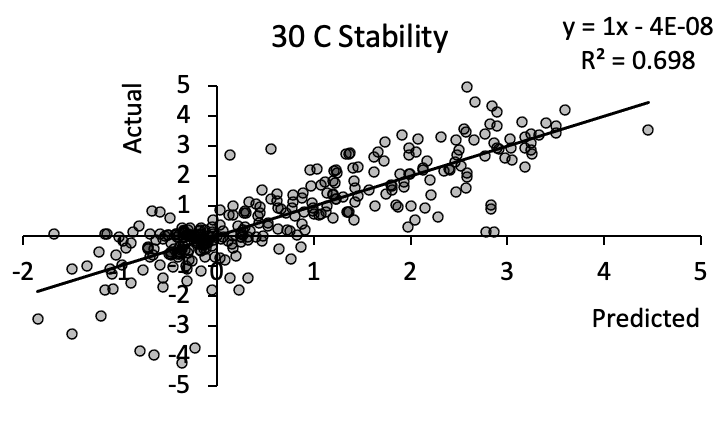

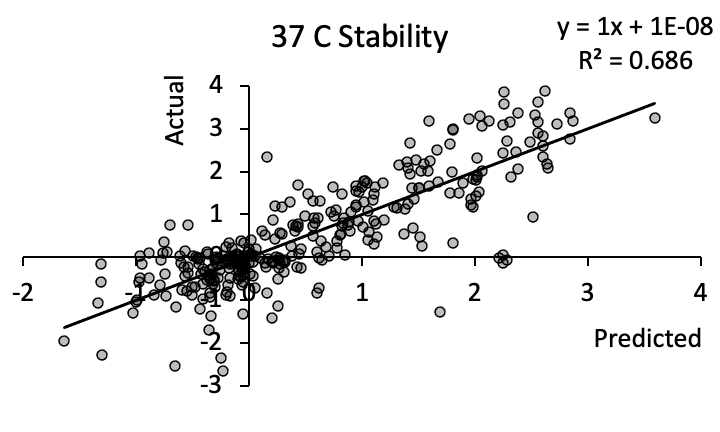


**I** 10 20 30 40 50
 IVGGSDSREG AWPWVVALYF DDQQVCGASL VSRDWLVSAA HCVYGRNMEP

* * * Q -* + -*- ** + * * + *
 60 70 80 90 100
 SKWKAVLGLH MASNLTSPQI ETRLIDQIVI NRHYNKRRKN NDIAMMHLEM
 * * * * + *--* P * * * * + ++
 110 120 130 140 150
 KVNYTDYIQP ICLPEENQVF PPGRICSIAG WGALIYQGST ADVLQEADVP
 * -S ** -+ +-+* + - -- * **
 160 170 180 190 200
 LLSNEKCQQQ MPEYNITENM VCAGYDAGGV DSCQGDSGGP LMCQENNRWL
 - * + - * * +* * - *- * * + -
 210 220 230

LAGVTSFGYQ CALPNRPGVY ARVPRFTEWI QSFLH

- - * * +

**Figure S5. Partial least squares regression models for the effects of mutations on EK_L_ variant properties.**

**A-D: Coefficients for each mutation for each response.** Mutations are ordered by significance according to their corresponding variable importance in projection (VIP) values from significant (blue, 6.3-1.0) to less significant (**red**, 1.0-0.6). Mutations with VIP<0.6 are not shown.

**E-H: Comparison of predicted responses to actual responses for the 321 variants.**

Plots represent each of the four responses of: (**A,E**) total activity when expressed at 30 °C; (**B,F**) total activity when expressed at 37 °C; (**C,G**) residual activity after pre-incubation at 50 °C for 2 hours, when expressed at 30 °C; (**D,H**) residual activity after pre-incubation at 50 °C for 2 hours, when expressed at 37 °C.

**I:** **Location and best impact from all mutated sites accumulated in the 321 sequenced variants.** + Sites with beneficial mutations (positive PLS coefficients, VIP>0.5) are shown as "+". Sites with deleterious mutations (negative PLS coefficients, VIP>0.5) are shown as "-", and the remaining sites (VIP<0.5) are shown as "*". Only the mutation with the most beneficial impact is reported where multiple possible mutations were observed at a given residue. Consensus mutations V15Q, R82P and C112S denoted separately as Q, P, S respectively.

***SDS-PAGE densitometry for selected evolved variants***

SDS-PAGE densitometry was carried out with 12.5 % Tris-Glycine pre-cast gels (Invitrogen Ltd.). Protein samples were prepared by first mixing 50 μL sample with 50 μL 2x Laemli sample buffer and then heated to 99 °C for 15 minutes before cooling to room temperature. 5 μL of sample was loaded into each well with 5 μL of PageRuler Prestained Protein Ladder (Fermentas Life Sciences) as a marker. For clarified lysate analysis, 5 μL/well of 20-fold concentrated clarified lysate samples were loaded whereas 20μL/well of Ni-affinity purified fractions and STI-affinity purified fractions were loaded. Additionally, 5 μL/well of 0.125 mg/mL BSA and 0.03125 mg/mL BSA were loaded as reference protein standards for comparison purposes. The protein gels were run with 1x Novex Tris-Glycine buffer (Thermo Fisher Scientific, UK) at 125 V for 120 minutes in XCell SureLock Mini-Cell (Invitrogen, UK) electrophoresis chambers. Gels were stained in sufficient volume of Coomasie blue (50% methanol, 10% acetic acid, and 0.5 g/L brilliant blue) for 30 – 45 minutes. Following, gels were de-stained in 100mL of de-staining reagent (40% methanol and 10% acetic acid) for 20-30 minutes and repeated as required. Gels were either visualised using a Gel-Doc IT Imaging System (UVP, UK) or visualized using a scanner after drying using a gel-drying kit.


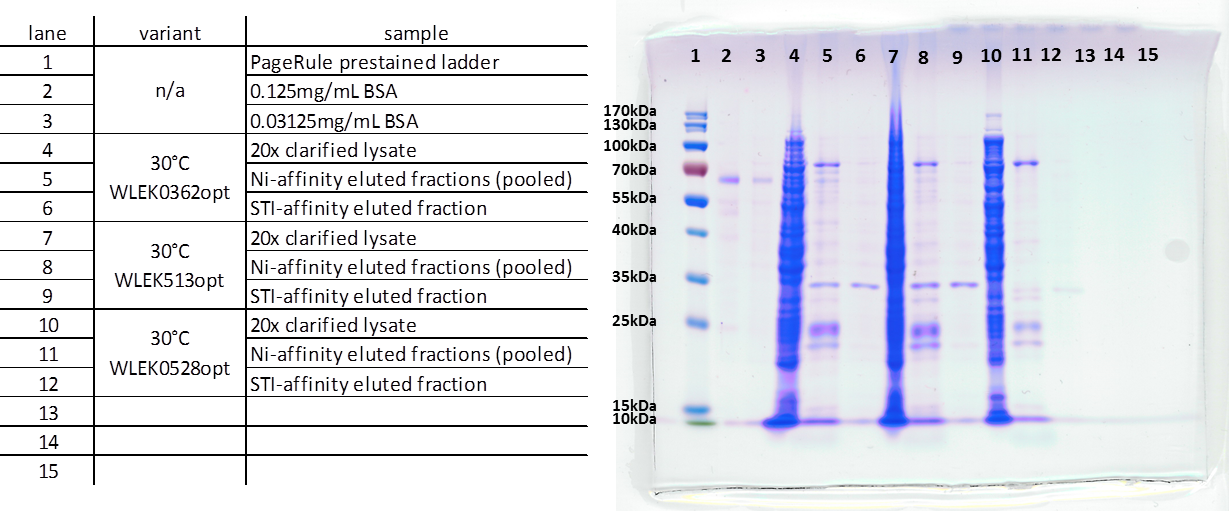


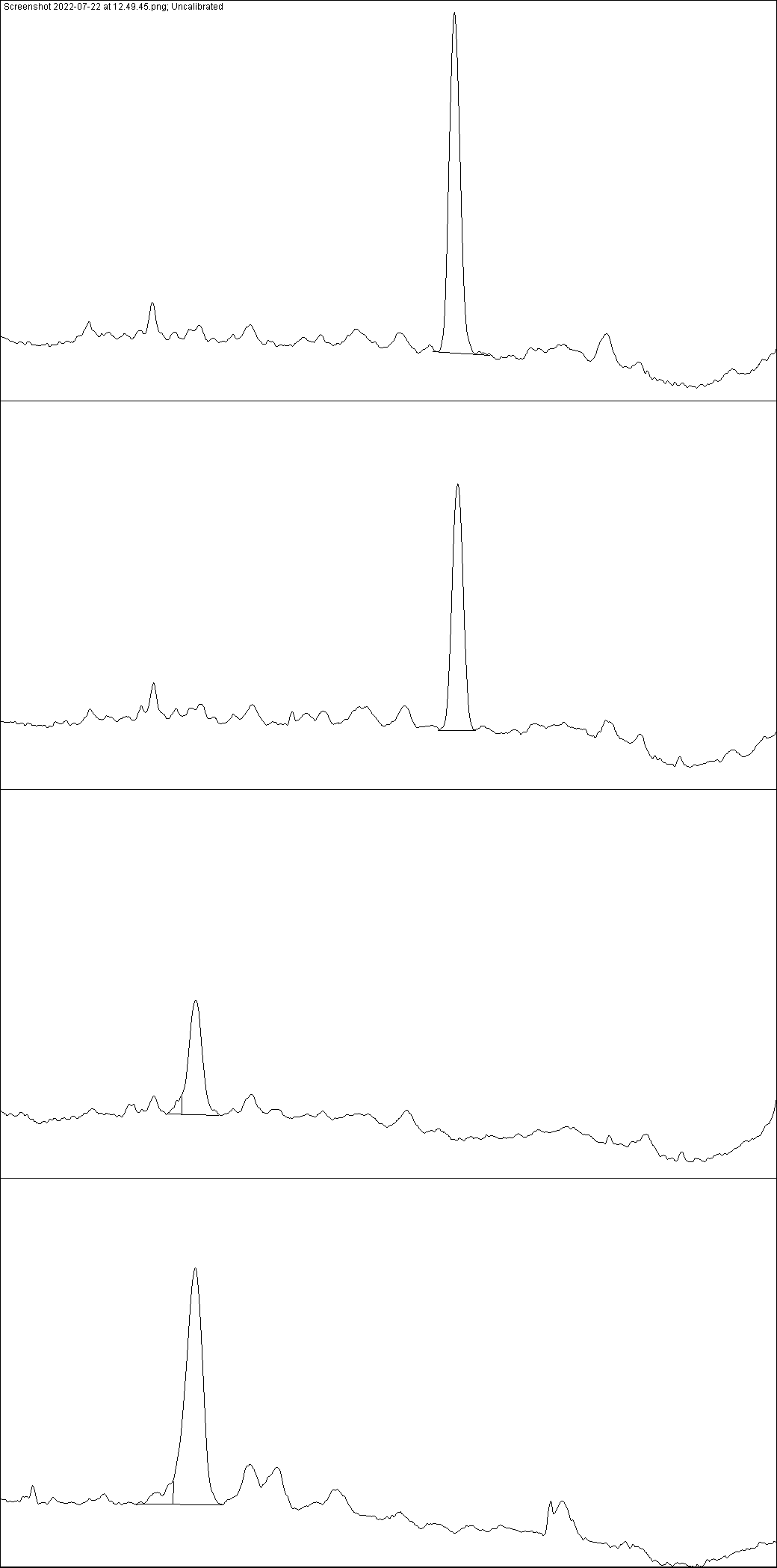


lane 9

lane 6

lane 3

lane 2

Figure S6. Example SDS-PAGE analyses of EK_L_ variants expressed at 30 °C. WLEK0362opt, WLEK0513opt, WLEK0528opt protein samples include 20-fold concentrated clarified lysates, pooled Ni-affinity elution fractions, and the STI-affinity elution fraction with highest EKL activity. The purified EK_L_ protein is in the band at just below the 35 kDa marker. Densitometry (see peak plots obtained using Image J in order of lanes 9, 6, 3, and 2), confirmed EK_L_ expression levels of at least 1.3 mg/L (WLEK0513) and 0.9 mg/L (WLEK0362) based on total purified material, comparable to the 1.4 mg/L (WLEK0513) and 1.3 mg/L (WLEK0362) measured via enzyme activity. A single gel is shown within a single unedited image, other than adjusting brightness and contrast evenly for the whole image.


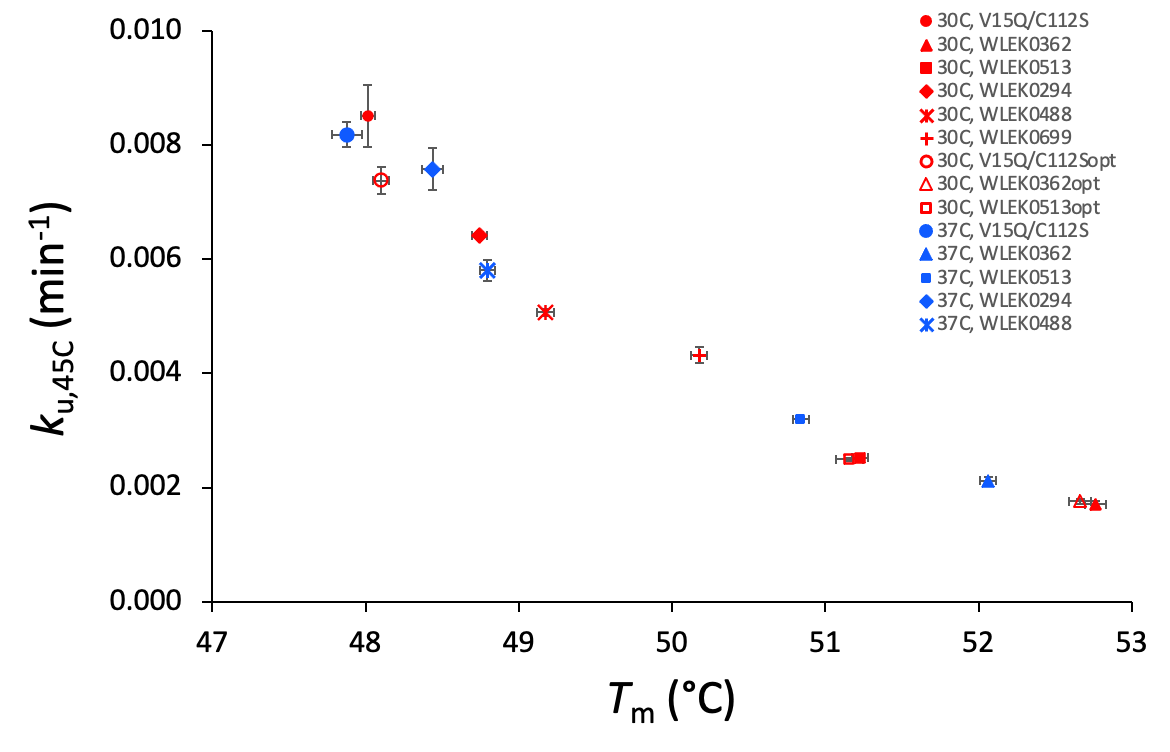


Figure S7. Relationship between unfolding rates of EK_L_ variants at 45 °C and their transition midpoints for thermal denaturation (*T*_m_), expressed at either 30 °C (red) or 37 °C (blue). Labels of individual variants are shown, while codon-optimised variants are shown with open symbols. Thermal denaturation transition mid-points, and unfolding rates at 45 °C (*k*_u,45°C_), were obtained for 0.1 mg mL^-1^ purified EK_L_ variants in 70 mM Tris-HCl, 50 mM NaCl, pH 8.4, and measured in triplicate from intrinsic fluorescence. Error bars are standard errors.

**A**


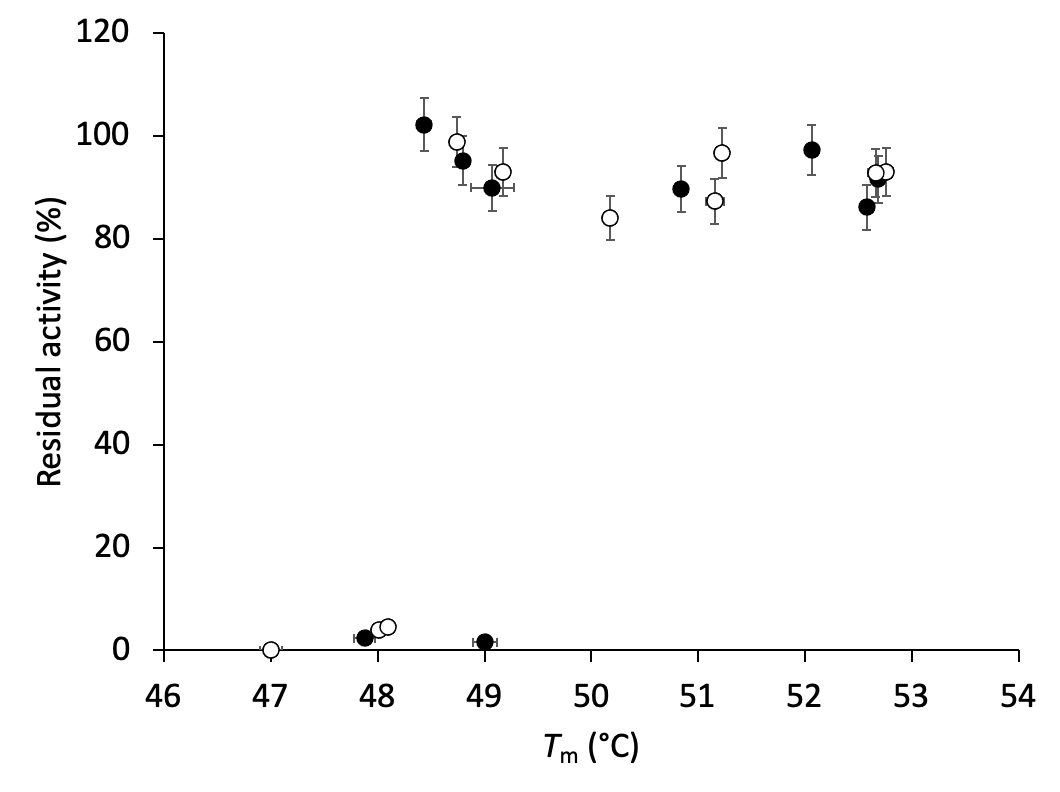


**B**


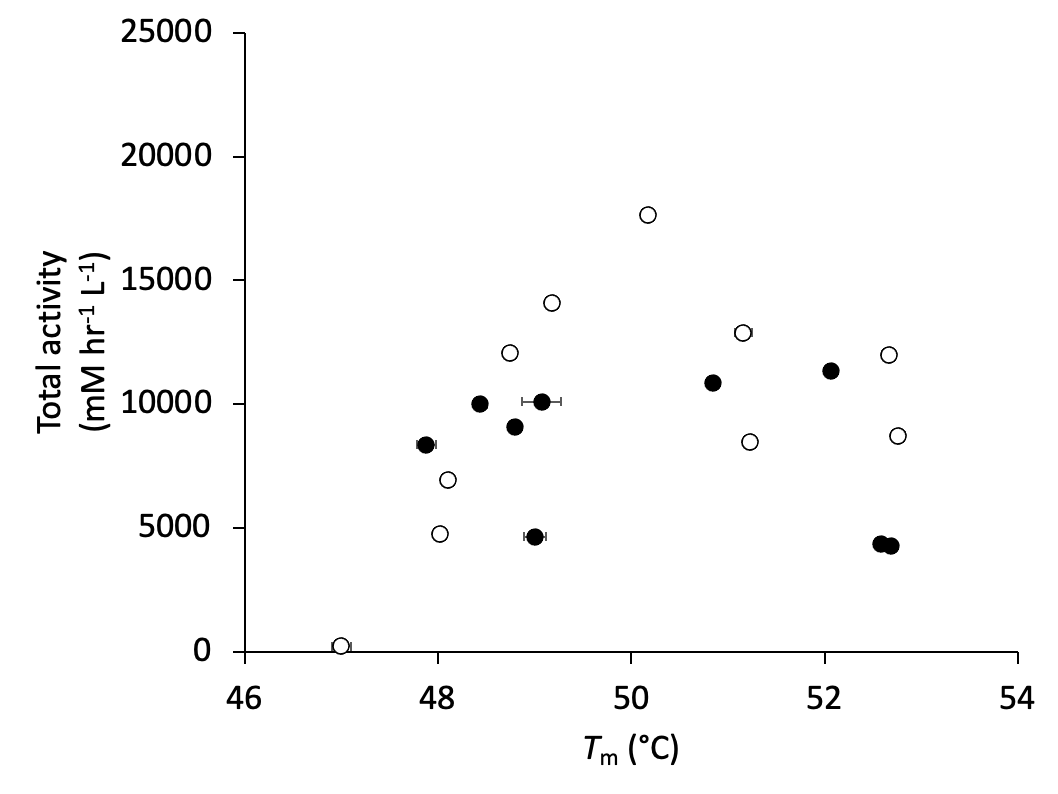


**Figure S8. Relationship between *T*_m_ for purified variants and their stability and total activity measured in clarified lysates.** **A**) Stability as measured from residual activity after incubation for 2 hours at 50 °C. **B**) Total activity measured from clarified lysates with 0.0625 mM substrate. Variants were obtained by expression at (⭘) 30 °C or (⚫) 37 °C. All error bars are standard errors of the mean.
